# Engineering a dysbiotic biofilm model for testing root caries interventions through microbial modulation

**DOI:** 10.1186/s40168-024-01862-5

**Published:** 2024-08-06

**Authors:** Naile Dame‐Teixeira, Reem El-Gendy, Andressa Souza de Oliveira, Cleonice Andrade Holanda, Luiz Antonio Soares Romeiro, Thuy Do

**Affiliations:** 1 Department of Dentistry, School of Health Sciences, University of Brasilia, Brasilia, Brazil; 2 Division of Oral Biology, School of Dentistry, University of Leeds, Leeds, UK; 3 Department of Oral Pathology, Faculty of Dentistry, Suez Canal University, Ismailia, Egypt; 4 Department of Pharmacy, School of Health Sciences, University of Brasilia, Brasilia, Brazil

**Keywords:** Dysbiosis, Oral microbiome, Dental caries, Root caries, In vitro model, Biofilm, Plant extracts

## Abstract

**Background:**

This study aimed to engineer and optimise a dysbiotic biofilm model to develop in vitro root caries for investigating microbial modulation strategies. The model involved growing complex biofilms from a saliva inoculum collected from four volunteers using two strategies. In the first strategy (“pre-treatment strategy”), bovine root slabs were used, and two natural compounds were incorporated at time 0 of the 10-day biofilm experiment, which included sucrose cycles mimicking the cariogenic environment. In the second strategy (“post-treatment strategy”), mature biofilms were grown in a modified Calgary biofilm device coated with collagen and hydroxyapatite for 7 days and then were exposed to the same natural compounds. The metatranscriptome of each biofilm was then determined and analysed. Collagenase activity was examined, and the biofilms and dentine were imaged using confocal and scanning electron microscopy (SEM). Mineral loss and lesion formation were confirmed through micro-computed tomography (μ-CT).

**Results:**

The pH confirmed the cariogenic condition. In the metatranscriptome, we achieved a biofilm compositional complexity, showing a great diversity of the metabolically active microbiome in both pre- and post-treatment strategies, including reads mapped to microorganisms other than bacteria, such as archaea and viruses. Carbohydrate esterases had increased expression in the post-treated biofilms and in samples without sugar cycles, while glucosyltransferases were highly expressed in the presence of sucrose cycles. Enrichment for functions related to nitrogen compound metabolism and organic cyclic component metabolism in groups without sucrose compared to the sucrose-treated group. Pre-treatment of the roots with cranberry reduced microbial viability and gelatinase (but not collagenase) activity (*p* < 0.05). SEM images showed the complexity of biofilms was maintained, with a thick extracellular polysaccharides layer.

**Conclusions:**

This root caries model was optimized to produce complex cariogenic biofilms and root caries-like lesions, and could be used to test microbial modulation in vitro. Pre-treatments before biofilm development and cariogenic challenges were more effective than post-treatments. The clinical significance lies in the potential to apply the findings to develop varnish products for post-professional tooth prophylaxis, aiming at implementing a strategy for dysbiosis reversal in translational research.

Video Abstract

**Supplementary Information:**

The online version contains supplementary material available at 10.1186/s40168-024-01862-5.

## Background

Despite the high prevalence of untreated dental caries [1], people are retaining more of their natural teeth as they age. This fact is followed by a rising incidence of root caries [2], which is a significant oral health concern and challenge posed by the progressive global aging of society. New approaches to the prevention and treatment of root caries are required, especially for aged and systemically compromised patients. A better understanding of root caries etiological mechanism, especially the interactions between dysbiotic microbial biofilms and host tissues, is essential for the development of new therapeutics. For instance, pro- and prebiotics have the potential to influence oral microbiota for promoting oral health and could be evaluated for their impact on microbial modulation on root caries dysbiotic biofilms. They could serve in the future as complementary components to root caries treatment and prevention strategies in combination with fluoride.

Exposure of dental roots to the oral environment significantly increases their risk of developing caries, which differ from enamel surfaces in several ways [3]. Root surfaces have a different organic and inorganic composition and morphology which make them more susceptible to biofilm accumulation and caries [3]. The acidic environment resulting from dietary sugar fermentation favours the growth and proliferation of aciduric and acidogenic bacteria [4]. As the dentine is exposed and collagen matrix become accessible for catabolism through the saliva or gingival fluid, proteolytic Gram-negative species can be selected [5], especially when root caries lesions extend beneath the gingival margin [6]. These species cohabit and act collaboratively to degrade the inorganic and organic components of the dental tissues, avoiding direct competition for specific nutrients (available in dietary sugars, saliva and gingival fluid). This fosters the establishment of compositionally and functionally diverse microbial communities on root surfaces [5]. The fact that root surfaces contain a considerable amount of organic material, as well as the presence of periodontal pathobionts with strong proteolytic activity in root surfaces biofilms [6], supports the theory that the microbiota might also play a role in the proteolytic stage of lesions formation [4, 7].

Designing a simple and reproducible laboratory-based method that accurately resemble a dysbiotic root caries biofilm is a crucial step in advancing novel prophylactic, diagnostic and therapeutic strategies targeting microbial functions. Various experimental models, including in vitro, ex vivo and human in situ models, have been employed to investigate the role of the microbiome in dental caries [8]. The ability to reproduce the biofilm community in the laboratory may increase the validity of the system to model microbiological events during caries lesion development [8, 9]. Despite achieving extensive demineralization with these methods mimicking cariogenic conditions, it is essential to recognize that natural biofilms are intricate ecosystems that cannot be fully studied when disassembled into their individual components. While simple methods using one of few microorganisms have significantly contributed to our understanding of certain factors contributing to cariogenicity, we must approach the extrapolation of these findings to real-life in vivo situations with caution and consider appropriate limitations.

This study aimed to engineer and optimize a dysbiotic root caries biofilm model for investigating microbial modulation. This model should be appropriate to demonstrate the potential of compounds in modulating root surface biofilm, and impacting root caries progression. Here, we investigated the effect of natural substances when used during biofilm development, or on a mature biofilm. In this context, we tested a phenolic lipid extracted from cashew nutshell liquid, which offers a combination of sustainability and bioactivity. This compound was previously shown to significantly reduce bacterial growth and collagenase activity [10]. Additionally, a cranberry extract was tested due to its anti-adhesion properties [11], antimicrobial capacity against oral biofilms [12] and potential as collagen cross-linker [13].

## Materials and methods

This study was reported according to the CRIS guidelines (Checklist for Reporting *in-vitro* Studies) [14].

### Study design

**Fig. 1:**
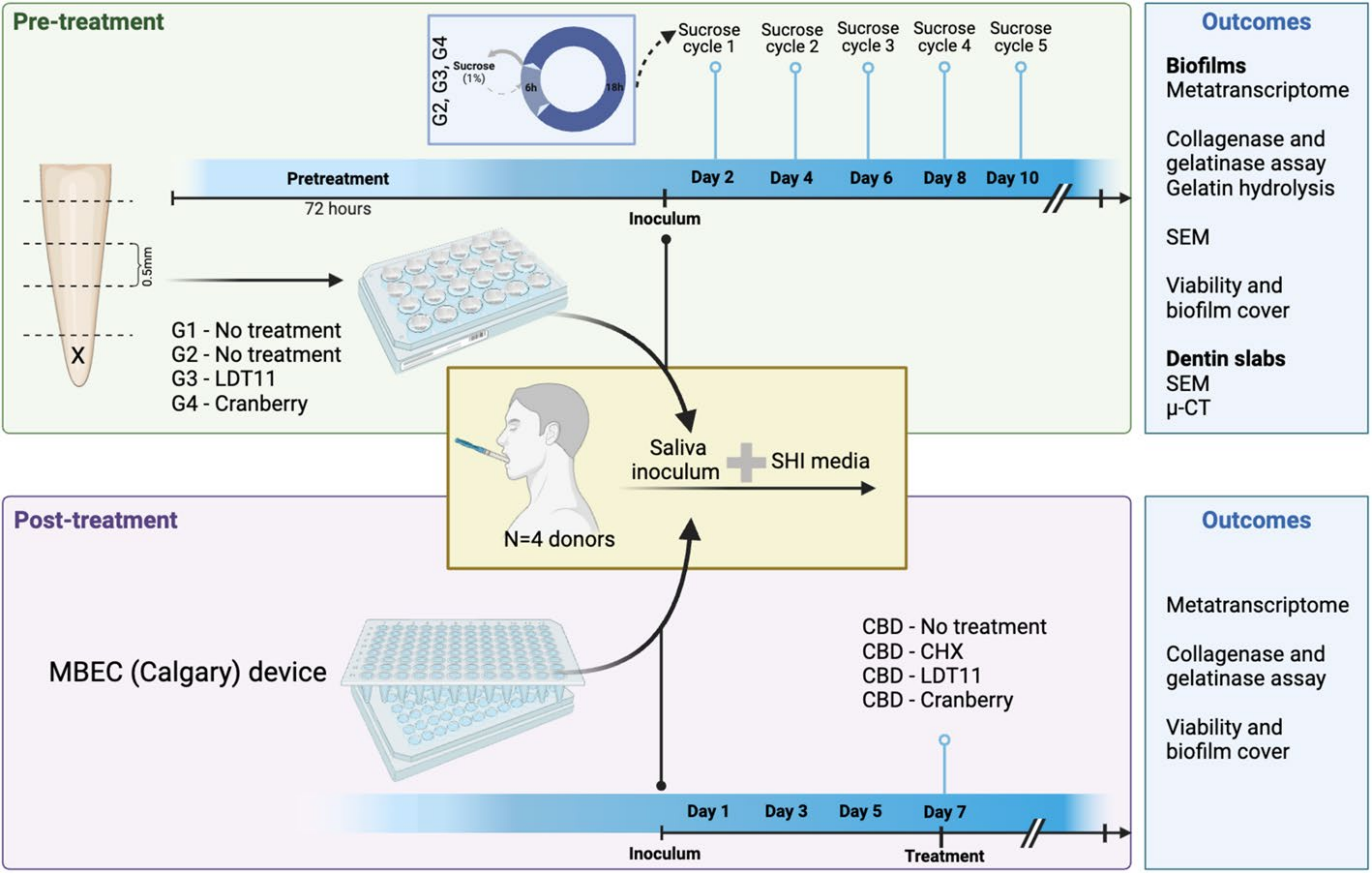
Experimental design. Two strategies were used: Pre-treatment (using bovine slabs and root caries formation) and Post-treatment (using a Calgary Biofilm Device to treat mature biofilms)

The experimental setup is illustrated in Fig. 1, showcasing the two different strategies employed. In the first strategy (“pre-treatment”), bovine root slabs were utilized, and test compounds were applied to the dentine slabs followed by cultivation for 10 days, with sucrose cycles simulating a cariogenic environment. This approach aimed to assess the compounds’ ability to modulate biofilms as pre-treatments to dentine and evaluate their potential for reducing lesion size compared to untreated controls. This pre-treatment strategy mimics the application of varnish products during dental treatments.

The second strategy employed “post-treatment” exposure. Mature biofilms were first cultivated in a Calgary Biofilm Device (CBD; MBECTM Assay System, MBEC Biofilms Technology Ltd., Calgary, Alberta, Canada) for 7 days. Subsequently, they were exposed to the test compounds. This approach aimed to assess the anti-collagenase and antimicrobial activity of the test substances against established mature biofilms, mimicking the use of mouthwashes.

### Samples

#### Initial inoculum and selection criteria for saliva donors

Four saliva donors were instructed to refrain from drinking and eating for 2 h. Non-stimulated saliva samples were collected through passive drooling and kept cool on ice to be delivered to the laboratory within an hour of collection. The following inclusion criteria were used to recruit the saliva donors: adults who self-reported either a caries experience (at least one tooth being restored or extracted due to caries) or a history of periodontal diseases (prior occurrence of gingivitis or periodontitis). This selection criterion was employed due to the similarity in microbial composition between individuals with inactive disease and those with active disease, as opposed to individuals without any history of caries [15, 16] or periodontal diseases [17]. Individuals with any history of diabetes, hypertension, arthritis, cancer, pregnant/lactating woman; who are using any kind of mouthwash, or used antibiotics in the last 3 months were excluded.

#### Description of devices preparation

##### Pre-treatment

Freshly extracted bovine incisors (*N* = 9) were cleaned of soft tissues and stored in fresh 0.5% chloramine for 48 h, at 4 °C. Then, stored in a humid environment (phosphate buffered saline- PBS) at 4 °C for no more than a month [18]. Following this disinfection protocol, roots were separated from the crowns under running water using a low-speed diamond saw (3000 rpm; Accutam, Struers). Then, four ~ 0.5 cm slices (transverse sections at axial plane) were sectioned from roots from cervical to apical direction, the first cut was positioned approximately 2 mm below the cement-enamel junction. The apical area was excluded. The entire buccolingual thickness of the root slices were maintained. The lingual surface of each slab was ground mechanically to obtain a flat surface. After preparation, specimens were affixed on the lid of a 24-well plate, leaving the buccal surface free. A post-preparation disinfection protocol with fresh 0.5% chloramine was performed for 48 h. In order to remove any chloramine residue from the specimens, a washing step was performed with sterile dH_2_O at 4 °C for 2 h [18], and storage at 4 °C in humidity (sterile PBS) for 1 week.

Randomization and allocation concealment of the slabs in each group were performed as follows: sequence generation was performed using random numbers tables generated for each tooth. Each tooth had its four slabs randomly assigned in four groups (1 slab per tooth in each group). The same investigator (ND-T) generated the randomisation sequence, sample collection and allocation to groups. Blinding was not feasible.

##### Post-treatment

To better mimic the root dentine biofilm and promote bacterial adhesion through collagen-binding proteins, we adapted the CBD by coating the hydroxyapatite pegs with a collagen coating solution (Sigma-Aldrich) to form a layer of collagen binding proteins. This modification allowed us to investigate not only adhesins but also other proteins involved in bacterial adhesion. CBD pegs were coated overnight with 200 μL of collagen coating solution (Sigma, concentration of 100 μL/0.32 cm^2^), at 25 °C, 65 rpm for 2 h. The pegs were then washed with sterile PBS.

For both strategies, another step with saliva coating was performed. The saliva was previously prepared by adding 2.5 mM dl-dithiothreitol, 50% of PBS and then filter-sterilised (filter membrane 0.22 µm pore size) to eliminate all contaminants and cells, leaving only the salivary proteins to form the pellicle at this stage. CBD and dentine specimens were coated with saliva for 1 h [19] at 37 °C; 65 rpm. Then, specimens were immersed in a proportion of 10% of initial inoculum added to SHI medium (proteose peptone, trypticase peptone, yeast extract, potassium chloride, sucrose, haemin, vitamin K, urea, arginine, mucin, blood, *N*-acetylmuramic acid-NAM) [20], which sustains the initial inoculum microbiota [21], and incubated in anaerobiosis and at 37 °C.

### In vitro microbial modulation strategies

#### Pre-treatment of the dentine slabs

Dentine slabs from one group received pre-treatment with the anacardic acid-derivative LDT11 at 100 μg/mL. For details of the compound synthesis and chemical structure, see [22]; for details regarding its potential usefulness in Dentistry, see [10]. Another group of dentine slabs was pre-treated with 100 μg/mL of cranberry extract (commercially available *Vaccinium macrocarpon* extract 36:1, Health4All purest, Blackpool, UK, batch 307). Before the experiment, specimens were completely immersed in the compounds for 72 h to assure the substances penetration into the dentine. Control groups were kept immersed in PBS [23].

After the incubation of the dentine slabs with saliva inoculum for 24 h, a simulation of the demineralization-remineralization process was conducted through 24-h cycles: 6 h with SHI media supplemented with 1% sucrose followed by 18 h with only SHI media (protocol modified from [24]). This cycle was repeated 5 times (every 24 h during the initial 7 days and every 48 h during the subsequent 7 days).

For this experimental strategy, the groups were described as: negative control with high pH (G1; no inoculum, no pre-treatment), positive control with low pH (G2 + ; with inoculum, no pre-treatment, with sucrose cycles); positive control with high pH (G2 − ; with inoculum, no pre-treatment, no sucrose cycles), pre-treatment with LDT11 (G3; with inoculum, with LDT11 pre-treatment, with sucrose cycles); pre-treatment with cranberry (G4; with inoculum, with cranberry pre-treatment, with sucrose cycles). Pre-treatment strategy groups are summarised in the Supplementary Table 1.

#### Post-treatment strategy

The CBD device was employed to cultivate mature biofilms for 7 days. Subsequently, the pegs with the biofilms were subjected to treatment with the compounds. Chlorhexidine (CHX) was used as the positive control and PBS as the negative control. The groups were categorized as follows: CBD (Negative control treated with PBS), CHX (Positive control with CHX at 100 μg/mL), CBD_LDT11 (Anacardic acid-derivative LDT11 at 50 or 100 μg/mL) and CBD_Cran (Cranberry at 50 or 100 μg/mL). To determine these concentrations, we first conducted a pilot study to observe the dose–response curve for planktonic microbes. This was followed by a series of biocompatibility experiments on monolayer dental pulp cells and unispecies biofilm models. We then selected the concentrations that demonstrated the highest impact on collagenase inhibition, antimicrobial activity and biocompatibility [10].

### Outcomes

The biofilms’ metatranscriptome was the main outcome of the study. Their antimicrobial activity and collagenase/gelatinase activity were also measured, as described below. In the pre-treatment group, mineral loss from dentine slabs was obtained via micro computed tomography (μ-CT).

### pH monitoring

The pH of the media was monitored using a calibrated pH meter electrode: within the pre-treatment strategy, daily pH measurements of the media were taken, immediately before introducing sucrose and after the 6-h mark. In the post-treatment strategy, throughout the process of biofilm formation, pH measurements were taken in triplicate each day.

### Metatranscriptome of biofilms

An aliquot of the harvested biofilms at the end of both strategies was mixed separately with RNAprotect, centrifuged and pellets stored at – 80 °C for further RNA extraction and sequencing. Total RNA yields ranged from 334.9 to 8526.3 ng (502.0 ± 118.8 ng). From the post-treatment analysis, only the groups exposed to one concentration (100 μg/mL) of the treatments was chosen. Total RNA samples were extracted from biofilms using the PowerFecal kit (QIAGEN), and used for library preparation. Briefly, all samples were checked for quantity and quality by agarose gel electrophoresis (RNA degradation and potential contamination), Nanodrop (tests RNA purity), Qubit (RNA concentration) and Agilent 2100 (RNA integrity). Then, the mRNA was purified including rRNA depletion and mRNA fragmentation, cDNA was synthetized, end repair and adaptor addition were performed and the fragments were enriched by PCR. The libraries were sequenced using Illumina (Novogene Co., Cambridge, UK).

Bioinformatic analysis was performed after removing low quality reads from raw data for analysis. First, the reads were assembled to conduct species classification analysis, and gene expression abundance analysis. Alignment of Unigenes with Bacteria, Fungi, Archaea and Viruses sequences extracted from NCBI’s NR database using DIAMOND software. Then homologous gene cluster analysis (eggNOG), carbohydrate enzyme analysis (CAZy) and other functional annotations were performed. Comparative analysis between groups was made, such as cluster analysis, PCA analysis and functional difference between samples (Novogene Co., Cambridge, UK).

### Antimicrobial characterization and biofilm thickness

Biofilms grown on dentine slabs (pre-treatment) or pegs (post-treatment) were incubated with Filmtracer Live/Dead Biofilm Viability Kit (Molecular Probes, Inc.) by incubation in a 1:1 ratio of SYTO9 and propidium iodide, as previously described [10], and then imaged within 24 h using a confocal laser scanning microscope (Leica Microsystems). 3D images were generated using Leica Application Suit X software, v. 3.5.7.23225 (LAS X, https://www.leica-microsystems.com/products/microscope- software/p/leica-las-x-ls/) and the Biofilm Viability Checker was used to calculate the area of green and red fluorescence [25]. This analysis was performed on biological duplicates for each group, with imaging conducted in two different fields for each group, resulting in a total of 180–253 fields analysed per group (pre-treatment) and 881–1002 (post-treatment). Rates of live to dead cells were calculated and compared between groups*.* In the same images and their fluorescent signal intensities, it was possible to calculate the biofilm thickness and the coverage area through examination of biofilm structure in relation to the spatial localization by choosing this option in the 3D viewer of the software.

### Collagenase/gelatinase activity of biofilms

To determine collagenase/gelatinase activity, the dysbiotic biofilms from both strategies were assayed in the EnzChek® Gelatinase/Collagenase Assay Kit using the DQ collagen type I (from bovine skin, fluorescein conjugate) and DQ gelatine as substrates (Molecular Probes, Inc.), following the supplier protocol.

In the pre-treatment strategy, biofilms were harvested with curettes after the end of the sucrose cycling period, washed twice with PBS and resuspended into 1 mL of PBS, from which 10 μL were added to the assay. The final collagen or gelatine concentration was 100 μg/mL. The assays were incubated in the experiment for 0, 2 and 24 h, in the shaker at room temperature. Fluorescence emission from the released fluorescent peptides at the collagen cleaving was monitored at 490–520 nm spectrum, at the Varioskan LUX Multimode Microplate Reader (ThermoScientific).

The gelatine hydrolysis of the biofilms from each the pre-treatment strategy was confirmed. Six replicates containing half of the biofilm formed in the dentine slabs were incubated anaerobically in nutrient agar (8 g/L) supplemented with gelatine (120 g/L) for 48 h, 72 h and 7 days. At these time points, the gelatine was assessed to ascertain either: partial hydrolysis (not all the gelatin used or a dense medium), total hydrolysis (completely liquid medium) or no gelatine hydrolysis.

For the post-treatment strategy, only the inhibition of gelatinase activity was performed as it was the single significant result in the pre-treatment strategy. Following 7-day biofilm formation and 30-min treatment with the natural compounds, CBD pegs containing the biofilms were immersed in DQ gelatine/buffer solution for enzymatic activity quantification. The fluorescence was measured at different time points (2 h, 7 h and 24 h) with incubation at room temperature (*N* = 7 pegs per group, experiment was carried out in duplicates).

### Biofilms morphology

Scanning electron microscopy (SEM) was employed to confirm morphologic modifications of the biofilm in different conditions. Samples were fixed with 2.5% glutaraldehyde for 12 h, followed by ethanol series dehydration. After gold sputter coating, samples were visualized with a scanning electron microscope. Biofilms were washed by dipping them in sterile PBS to remove loosely adherent cells.

### Confirmation of the root caries lesion formation

The outcome for the work on dentine slabs (pre-treatment strategy) was the presence or absence of the carious lesions confirmed by the mineral loss of the slabs through μ-CT scanning before and after the experimental model. The scans were performed using medium camera pixels with a special resolution of 10 μm and with Al + Cu filters (Skyscann, Bruker). Reconstruction was performed with the software interface (Nrecon). Differences between the reconstitute images before and after the experimental model and cariogenic challenges were created into the software and analyzed for the presence or absence of structure loss.

#### Sample size

Imaging (CSLM and μ-CT) and metatranscriptome analyses were performed in triplicates. SEM images were performed in one representative sample from each group in a descriptive analysis using different magnitudes. A sample size calculation was performed using the outcome “collagenase activity” (fluorescence values) of the enzyme control (*Clostridium histolyticum* collagenase) = 0.55, the average of oral bacteria (*Streptococcus mutans*, *Veillonella parvula*, *Veillonella dispar*, *Escherichia coli*, *Porphyromonas gingivalis*) = 0.43, and a standard deviation of 0.08, for a power of 80% and alpha of 5%. A sample size of *n* = 7 biofilms was considered enough for this outcome (http://powerandsamplesize.com/Calculators/Compare-k-Means/1-Way-ANOVA-Pairwise). A meaningful difference between groups was expected to be 10% in the microbial collagenase activity.

#### Statistical methods

Statistical analyses were performed using the GraphPad Prism 9 for Macbook. Statistical methods used to compare groups for pH: Mixed-models + Tukey, ANOVA + Dunnett’s (post-treatment main effect time). The antimicrobial and biofilm coverage were compared between groups using Kruskal–Wallis followed by the Dunn’s post hoc test.

## Results

### pH modulation within each experimental strategy

**Fig. 2:**
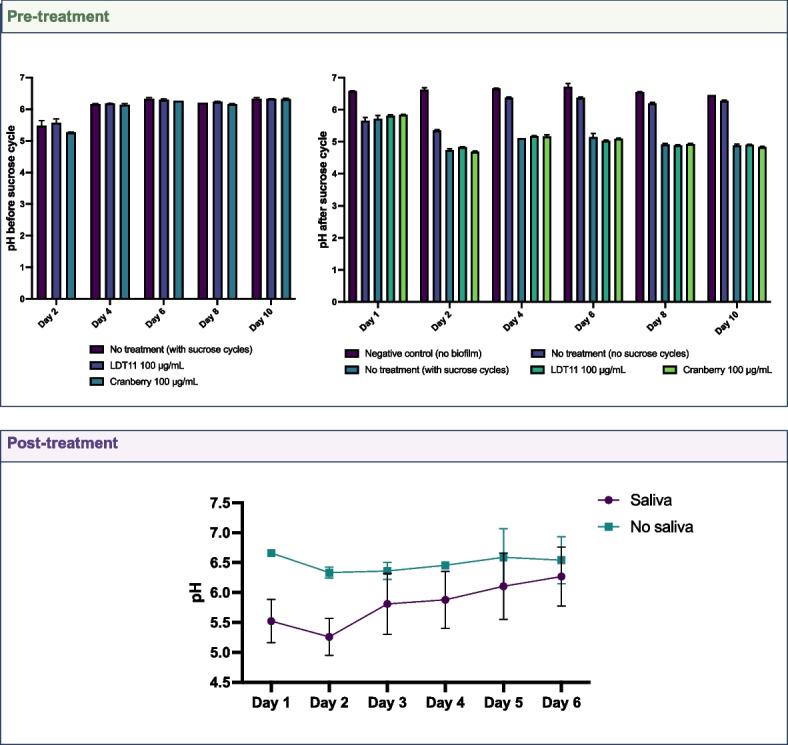
Measurement of the pH of the media. Pre-treatment strategy: pH was measured immediately before and 6 h after adding sucrose to the media in the cycling. Post-treatment strategy: pH was measured daily, before the media change. Experiment performed using a pH meter electrode. Statistical analysis: Pre-treatment, before sucrose cycles = multiple comparisons regarding time points. Pre-treatment, after sucrose cycles = ANOVA, Dunnett’s multiple comparisons against no treatment (with sucrose cycles). Post-treatment = Statistical model for the main effect time. Mixed-effect model; Tukey's multiple comparisons test

Figure 2 illustrates the pH change during the biofilm formation. For the pre-treatment strategy, the pH was measured before adding sucrose and 6 h after sucrose addition, confirming pH fluctuations in the cycles. Prior to exposure to sucrose cycling, the pH of the media in the cranberry-pre-treated dentine slab group was lower on day 2 when compared to day 4 (*p* = 0.0479), day 6 (*p* = 0.0211), day 8 (*p* < 0.0001) and day 10 (*p* = 0.0211), but recovered by day 4. In contrast, the untreated group (“No treatment, with sucrose cycle”) exhibited a higher pH by day 10 (*p* < 0.0001). The groups with cycles displayed lower pH levels compared to the control group without sucrose cycles, reaching the critical pH for root dentine demineralization. The group without treatment and not exposed to sucrose cycles increased the pH over time, remaining above the critical pH for root dentine demineralization at the end of the experiment. On day 1, no significant differences were observed between the groups, confirming the baseline pH similarity. However, the “no treatment (with sucrose cycles)” group had a significantly lower pH compared to the negative control (no biofilm) on day 2 (*p* = 0.0037), day 4 (*p* = 0.0046), day 6 (*p* = 0.0108), day 8 (*p* = 0.0027) and day 10 (*p* = 0.0209). This group also had a significantly lower pH than the “no treatment (no sucrose cycles)” group on day 2 (*p* = 0.0329), day 4 (*p* = 0.0176), day 8 (*p* = 0.0011) and day 10 (*p* = 0.0062) (Supplementary Table 2).

Measurements of the media pH were conducted daily for the post-treatment strategy. A clear and statistically significant pH elevation was observed following the third day of biofilm formation: day 1 vs. day 5 (*p* = 0.0008), day 1 vs. day 6 (*p* < 0.0001), day 2 vs. day 3 (*p* = 0.0017), day 2 vs. day 4 (*p* = 0.0004), day 2 vs. day 5 (*p* < 0.0001), day 2 vs. day 6 (*p* < 0.0001), day 3 vs. day 6 (*p* = 0.0016), day 4 vs. day 6 (*p* = 0.0079) (Supplementary Table 2). This phenomenon could potentially be attributed to the increased complexity of the biofilms and the attachment of late colonizers. This trend aligns with the observed pH increase over successive days in the untreated group of the pre-treatment strategy.

### Biofilms metatranscriptome–RNAseq

#### Sequencing output

The number of clean reads (filtered sequencing data) ranged from 3.78 × 10^7^ to 4.74 × 10^7^, and subsequent bioinformatic analysis is based on these reads. Clean bases ranged from 5.7G to 7G. The GC% content was lower in the saliva inoculum (43.36%) and higher (51.05%) in G2_3 (replicate of the pre-treatment strategy G2 + group–without pre-treatment, with sucrose cycles).

#### Microbial composition

**Fig. 3:**
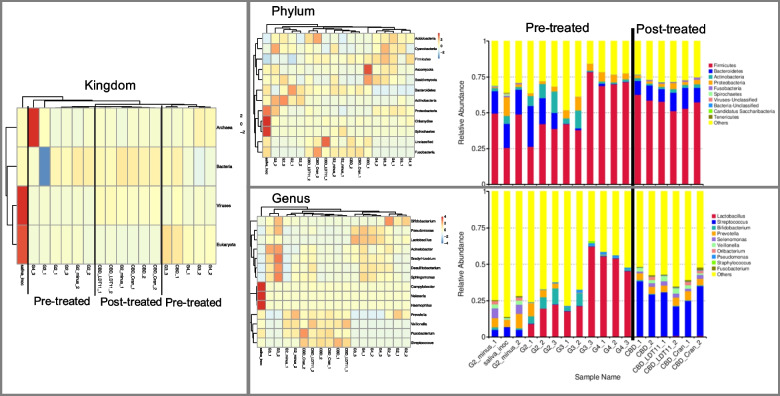
Taxonomic abundance clustering of the 35 most abundant taxa (Kingdom; phylum, and genus-level) within each sample. Samples were first selected based on species identification and abundance information. Taxa were then clustered based on both their taxonomic relationships and differences in abundance between samples. The clustering tree was constructed using the Bray–Curtis distance metric. On the right, the relative abundance distribution of each sample is shown, highlighting the top 10 taxa with the highest relative abundance at each taxonomic level. Remaining species are grouped as “Others”

As expected, there was a slight reduction in the number of active taxa within the laboratory culture when comparing the biofilms to the initial inoculum. The phyla *Chlamydiota*, *Spirochaetota* and *Proteobacteria*, as well as the *Saccharibacteria* oral taxon TM7x, were affected by the laboratory conditions and were found reduced in numbers following culturing. However, this diversity reduction was less noticeable in the biofilms grown on dentine slabs without treatment and without sucrose cycles (pre-treatment strategy, G2 − group). Nevertheless, the biofilm compositional complexity was confirmed, showing a great diversity of the active microbiome in both pre-treatment and post-treatment strategies, including reads mapped to microorganisms other than bacteria, such as archaea and viruses (Fig. 3).

The clustering tree based on Bray–Curtis distances struggled to differentiate between the biofilms from both strategies, particularly at higher taxonomic levels. At the genus level, the majority of samples subjected to sucrose cycles, regardless of pre-treatments, formed a clustered group. An exception was observed with two samples pre-treated with the anacardic acid LDT11. Conversely, all samples from the post-treatment strategy exhibited close clustering, implying that sucrose cycling significantly influenced sample composition. Taxa previously found in natural root caries lesions were the most active genus: *Lactobacillus, Streptococcus*, *Bifidobacterium*, *Prevotella*, *Selenomonas*, *Veillonella*, *Oribacterium*, *Pseudomonas*, *Fusobacterium*, *Campylobacter*, *Actinomyces*, *Bacteroides*, *Neisseria*, *Staphylococcus*, *Haemophilus*, *Desulfitobacterium* and *Treponema*. All samples that were exposed to sucrose cycles—pre-treated or not—massively increased the proportion of lactobacilli.

Concerning the non-bacterial content, the *Thaumarchaeota* and *Euryarchaeota* archaeal phyla were detected in the initial inoculum, with *Thaumarchaeota* exhibiting reads only in the groups subjected to sucrose cycles. On the other hand, reads affiliated with the *Crenoarchaeota* phylum were absent in the initial inoculum but were found in the groups treated with anacardic acid and cranberry, whether applied as pre or post-treatments, according to the relative abundance of unigenes. Virus reads were identified across all samples, and an unclassified virus phylum stood as the seventh most prevalent within the metatranscriptome across all conditions (Supplementary file 2, taxonomic analysis, unigene relative abundance table).

#### Differential gene expression and gene ontology term enrichment

**Fig. 4:**
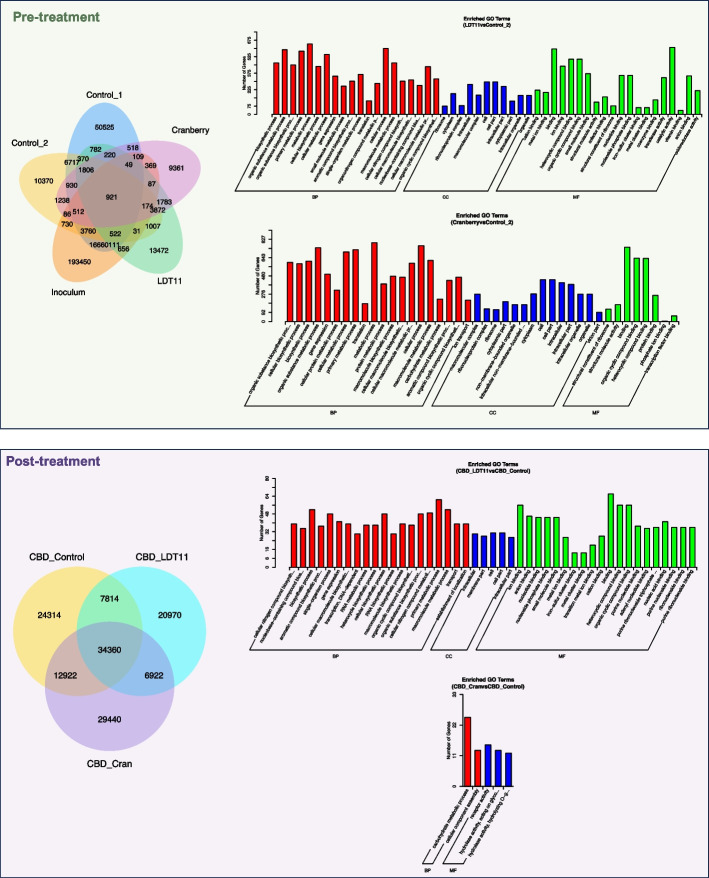
RNA-seq coexpression (Venn diagram) and differential expression enrichment (GO terms—Gene Ontology). Much less co-expression in the pre-treatment is observed (Control 1 = G2 − ; Control 2 = G2 +). In the post-treatment, all groups shared similar expression in 34,360 genes. Cellular components (CC); biological processes (BP); metabolic functions (MF)

There was lower co-expression (number of uniquely expressed genes shared between 2 groups) in the pre-treatment group (Fig. 4; Venn diagram): 921 genes sharing the same expression within all samples of the pre-treatment strategy, while there were 34,360 genes in the post-treatment groups. Analysis of differentially expressed genes revealed distinct expression patterns under different experimental conditions. Notably, the three groups exposed to sucrose clustered separately from those without sucrose addition, indicating a clear response to sucrose (Supplementary Fig. 1).

In the Gene Ontology (GO) terms enrichment analysis, both anacardic acid LDT11 and cranberry pre-treatments induced a higher level of gene expression alterations in biofilms: around 10 times more genes displayed significant differential expression in comparison to the post-treatment involving the same substances. In the post-treatment strategy, the anacardic acid derivative LDT11 provoked minimal changes in the cellular components of the mature biofilm but exerted substantial influence on biological processes and metabolic functions. Pre-treatment with anacardic acid LDT11 promoted changes in the metal binding in the biofilm growth in pre-treated with anacardic acid LDT11 when compared to controls without pre-treatment (> 500 genes with differential expression in each function). The same substance used as post-treatment did not change much the cellular components (CC) but did in the biological processes (BP) and metabolic functions (MF), although less than 60 genes had differential expression with the respective control without treatment. Of particular interest, the initial inoculum and the G2 − group (pre-treatment strategy without sucrose cycles) displayed no differentially expressed enriched GO terms. Similarly, the absence of enriched GO terms was observed when the biofilms harvested following the pre-treatment with anacardic acid LDT11 and cranberry were compared.

GO terms enrichment analysis of differentially expressed genes revealed that the control group (G2 −) exhibited enrichment for functions related to nitrogen compound metabolism and organic cyclic component metabolism compared to the sucrose-treated group (G2 +) (Supplementary Fig. 2). The branches represent hierarchical relationship (darker colour means higher enrichment degree of the biological process, cellular component or metabolic function).

#### Function annotation with EggNOG and carbohydrate metabolism

**Fig. 5:**
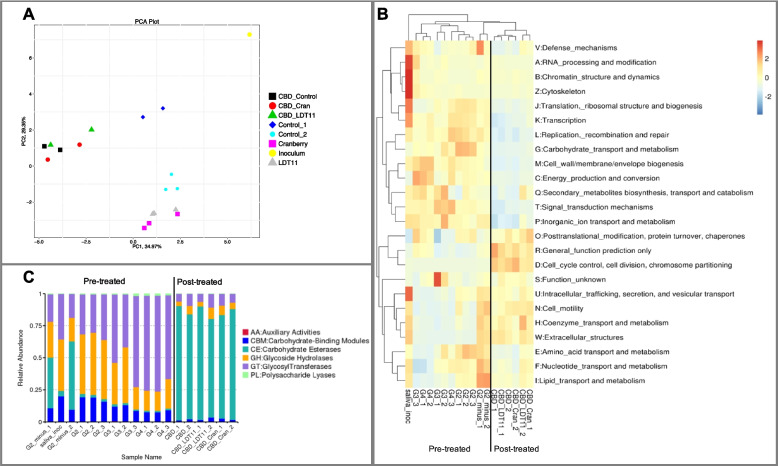
Function annotation with Evolutionary Genealogy of Genes Non-supervised Orthologous Groups (EggNOG) and Carbohydrate metabolism (CAZy). **A** Functional annotation with eggNOG – clustering PCA: pre-treatment groups clustered farther apart in the functional abundance distance matrix, indicating that pre-treatments had a more profound impact on biofilm metabolic profiles. **B** Heatmap (EggNOG): Biofilms growth over the dentine slabs without treatment, and exposed to sucrose cycles had more carbohydrate transporters. Post-treatment: do not change much the gene expression. **C** Static abundance chart on the first level of CAZy

The analysis of functional abundance distances indicated that, in terms of metabolism, the pre-treatment variants exhibited greater dissimilarity compared to the post-treatment ones (Fig. 5A). The heatmap (EggNOG database) on Fig. 5B shows that biofilm growth over the dentine slabs without treatment, and exposed to sucrose cycles had more carbohydrate transporters (G2 + group). In samples from the post-treatment strategy, no important dissimilarities were observed in the gene expression. The group not exposed to sucrose (G2 − group) had higher expression in amino acid, lipid, nucleotide and coenzyme transport and metabolism. Saliva inoculum presented higher expression defence mechanisms, RNA processing, translation, transcription, replication.

Enriched GO terms can be observed in Fig. 5C, showing that biofilm growth over the dentine slabs without treatment, and exposed to sucrose cycles had more carbohydrate transporters. Carbohydrate esterases (in green) had higher level of expression in the post-treated biofilms and the ones without sugar cycles, while glucosyltransferases (purple bars) were highly expressed in the pre-treated root slabs in the presence of sucrose, showing once more the cariogenicity of biofilms in our model.

We specifically observed the bacterial collagenases gene expression due to their importance to root caries biofilms. Out of the 32 annotated genes for collagenases U32, only four did not belong to *Prevotella* species. Interestingly, no reads were obtained for these genes in the anacardic acid LDT11 group, and just few reads in the cranberry group (Supplementary Fig. 3).

### Antimicrobial effectiveness and biofilm thickness

**Fig. 6:**
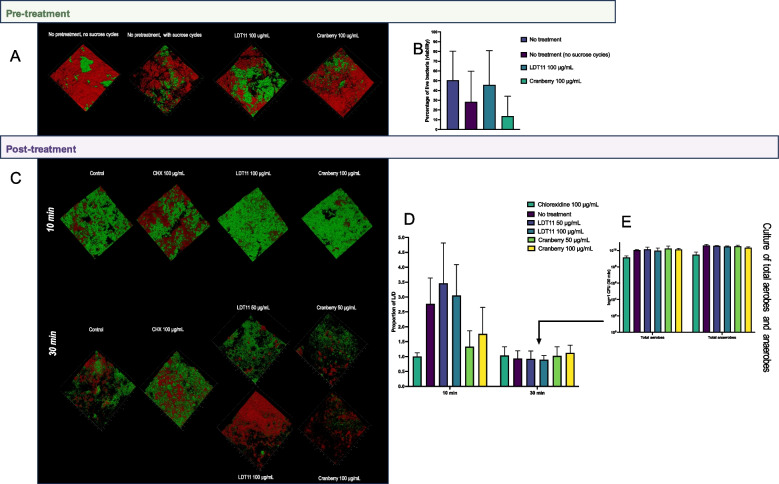
Antimicrobial activity of the compounds against complex biofilms when used as pre-treatment or post-treatment. **A** Confocal Light Scanning Microscopy (CLSM) 3D images for the pre-treatment strategy. **B** Proportion of viable cells (viability) measured with the biofilm viability checker using the CLSM images from a biological duplicate, with imaging conducted in two different fields for each group, resulting in a total of 180–253 fields analysed per group. Statistical analysis: Biofilm viability Checker; Kruskal–Wallis, Dunn’s–control “No treatment (sucrose cycles)”. **C** Confocal light scanning microscopy 3D images for the post-treatment strategy from a biological duplicate, with imaging conducted in two different fields for each group, resulting in a total of 881–1002 fields analysed per group. **D** Proportion of life and dead (L/D) cells in post-treated biofilms measured with the biofilm viability checker using the CLSM images. Statistical analysis: 2-way ANOVA, Tukey’s multiple comparison test. **E** Culture of the total aerobes and total anaerobes after 30 min of post-treatment. Statistical analysis: 2-way ANOVA, Tukey’s multiple comparison test

We assessed the antimicrobial effectiveness of the compounds in both pre- and post-treatment applications (Fig. 6). Figure 6A illustrates examples of confocal 3D images depicting significant areas of dead cells in both the cranberry pre-treatment and sucrose control groups. This was confirmed by the Kruskal–Wallis test, followed by Dunn’s multiple comparisons against the control “No treatment (no sucrose cycles)”, that shows that the pre-treatment with cranberry extract significantly reduced biofilm viability (*p* < 0.0001). LDT11 exhibited comparable cell viability to the control group with sucrose cycles (*p* = 0.1209), suggesting a potential mitigation of the pH effects within the biofilm. Similarly, the group with no pre-treatment and no sucrose cycles also exhibited diminished viability relative to the other groups, except for the cranberry-treated group (*p* < 0.0001) (Fig. 6B).

In the post-treatment strategy, following a 10-min incubation with the compounds, no noticeable changes were observed on the biofilm surfaces, with the exception of the positive control, chlorhexidine (CHX). However, after a 30-min treatment period, impacts were evident on the outer layers of the biofilms when exposed to higher concentrations of both anacardic acid and cranberry compounds (Fig. 6C). An examination of the internal layers of the biofilm revealed that, after a 10-min treatment, cranberry exhibited significantly stronger antimicrobial properties compared to anacardic acid LDT11, and their dosage effects appear to have opposite results: the proportion of live and dead cells was higher in LDT11 at 50 μg/mL compared to cranberry at 50 μg/mL (*p* < 0.0001), LDT11 at 50 μg/mL compared to cranberry at 100 μg/mL (*p* = 0.0013), LDT11 at 100 μg/mL compared to cranberry at 50 μg/mL (*p* = 0.0029) and LDT11 at 100 μg/mL compared to cranberry at 100 μg/mL (*p* = 0.0454). The no treatment group exhibited higher effects than cranberry at 50 μg/mL (*p* = 0.0479). LDT11 also showed less effect than chlorhexidine (CHX) (LDT11 at 50 μg/mL vs. CHX at 100 μg/mL—*p* < 0.0001, LDT11 at 100 μg/mL vs. CHX at 100 μg/mL—*p* = 0.0003), while cranberry did not differ from the positive control CHX, nor between its concentrations of 50 μg/mL and 100 μg/mL. Yet, this difference became less pronounced after a 30-min treatment, where no statistical differences between groups were observed (Fig. 6D, supplementary Table 3). However, the culture of total anaerobes and aerobes from the biofilms treated for 30 min demonstrated increased sensitivity, allowing for differences in the antimicrobial efficacy of CHX in comparison to the other groups (*p* < 0.0001) (Fig. 6E).

**Fig. 7:**
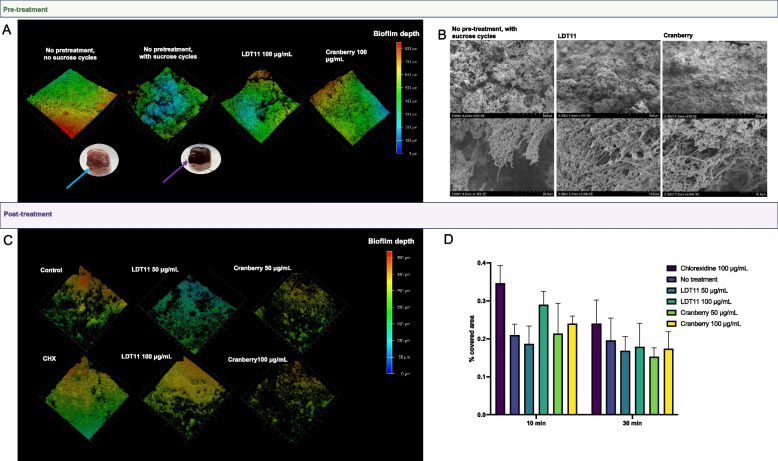
Analysis of the biofilm characteristics. Pre-treatment strategy: **A** Biofilms depth: the confocal microscopy used the same images from Fig. 6A and C, and their fluorescent signal intensities, to calculate the biofilm thickness and the coverage area through examination of biofilm structure in relation to the spatial localization by choosing this option in the 3D viewer of the Leica Application Suit X software, v. 3.5.7.23225 (LAS X, https://www.leica-microsystems.com/products/microscope- software/p/leica-las-x-ls/), and representative biofilm photographs (blue and purple arrows represents no pre-treatment groups, without and with sucrose cycles, respectively). **B** Scanning electronic microscopy (SEM) at two magnifications, including the control group without pre-treatment and with sucrose cycles, and the pre-treated groups with LDT11 and cranberry. SEM images magnification: top row = 5.00 kV, 4–5 mm × 100 SE, 500 µm for all images; bottom row = 5.00 kV, 4.5 mm × 1 50 k SE, 30.0 µm; 5.00 kV, 4.5 mm × 1 3.00 k SE, 10.0 µm, and 5.00 kV, 5 mm × 1 3.00 k SE, 10.0 µm. Post-treatment strategy: **C** Biofilm depth; **D** Biofilm Coverage area after post-treatment for 10 and 30 min. The experiment was conducted using 4 distinct pegs per group, with each peg subjected to imaging in two separate regions

In the pre-treatment strategy, the sucrose cycles contributed to the development of a less dense, more porous biofilm structure. This observation aligns with the expected presence of extracellular polysaccharides that provide structural integrity to this biofilm matrix (Fig. 7A and B). In the presence of sucrose cycles, the biofilms exhibited a brownish coloration (Fig. 7A, purple arrow), whereas in the absence of sucrose cycles, they appeared more yellowish (Fig. 7A, blue arrow).

Both the cranberry and anacardic acid LDT11 groups exhibited numerous voids within the biofilm structure within the 3D images of the post-treatment strategy (Fig. 7C). By quantifying the area covered by the biofilms (Fig. 7D), we showed that cranberry facilitated the detachment of cells (anti-biofilm effect) in the post-treatment strategy (cranberry 50 μg/mL vs. CHX control at 10 and 30 min; *p* = 0.0130 and *p* = 0.0078, respectively; mixed-effect analysis), but not in the pre-treatment strategy.

### Collagenase and gelatinase activity of biofilms

**Fig. 8:**
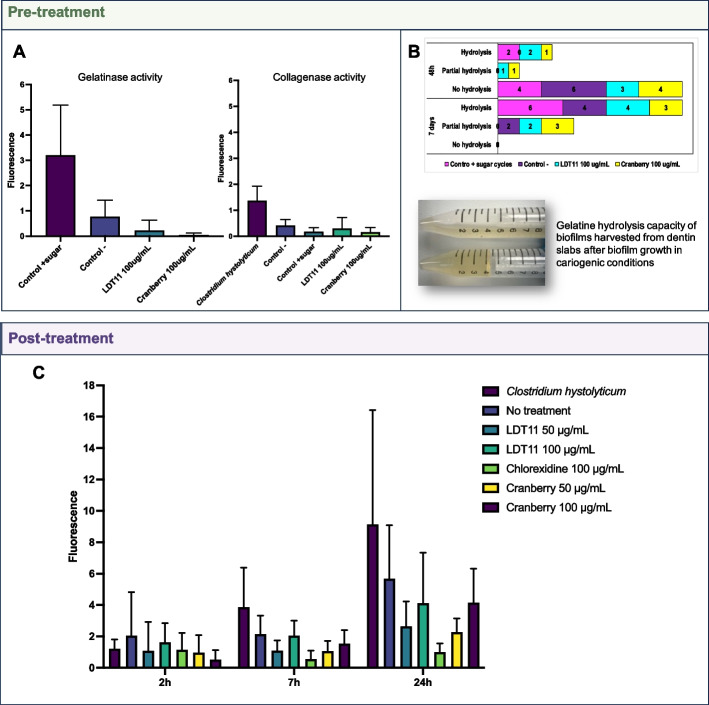
Collagenase and gelatinase activity of the biofilms with and without pre-treatments or post-treatments with the anacardic acid LDT11 and the cranberry extract. **A** Pre-treatment gelatinase and collagenase activity (EnzChek Gelatinase/Collagenase assay kit using the DQ collagen type I and DQ gelatine as substrates). Statistical analysis: 2-way ANOVA, Dunn’s multiple comparisons test. **B** Pre-treatment strategy: gelatine hydrolysis, assessed at two time points (48 h and 7 days). The bar graph indicates the number of replicates that were positive for hydrolysis. The image below the graph displays two tubes containing gelatine: the top tube shows positive hydrolysis with the gelatine in a liquid state, while the bottom tube shows negative hydrolysis with the gelatine remaining solid. **C** Post-treatment gelatinase activity (EnzChek Gelatinase/Collagenase assay kit using the DQ gelatine as substrate). Fluorescence was measured at three time points 2 h, 7 h and 24 h. Statistical analysis: 2-way ANOVA, Dunnett’s multiple comparisons test

Investigating the inhibition of collagenases as a targeted approach for the treatment and prevention of root caries is promising, especially considering the likely role of microbial collagenases in the second stage of lesion formation. In this study, we examined whether the LDT11 and cranberry could demonstrate the modulation of these biofilms by inhibiting collagenases. Figure 8 presents the outcomes of the collagenase and gelatinase inhibition assessments (Fig. 8A and C), along with the gelatine hydrolysis experiments (Fig. 8B). In the pre-treatment strategy, collagenase activity of biofilms exhibited lower levels when collagen type 1 was the substrate than gelatine. The *C*. *histolyticum* positive control exhibited higher collagenase activity compared to biofilms from the control group (no pre-treatment, with sugar cycle) (*p* = 0.0389), LDT11 at 100 μg/mL (*p* = 0.0499) and cranberry at 100 μg/mL (*p* = 0.0153). No significant differences were found between the control (no pre-treatment, with sugar cycle) and the compounds. Also, pre-treatment with cranberry yielded biofilms with reduced gelatinase activity (*p* = 0.0114) (Fig. 8A). This observation was subsequently verified through the gelatine hydrolysis experiment, where biofilms harvested from the dentine after 10 days of biofilm and lesion development were placed in gelatine-containing media for 7 days, with subsequent observation of liquefaction occurrence. The proteases found in biofilms taken from dentine slabs were able to completely hydrolyse gelatine (tubes show positive hydrolysis when the gelatine was found in a liquid state at each time point), except for a few replicates pre-treated with cranberry or anacardic acid LDT11, or in controls without sugar cycles, that partially hydrolysed gelatine. Among these, the cranberry-treated group displayed a larger number of replicates with partial hydrolysis, indicating that the gelatin was not completely liquefied (Fig. 8B). For investigating collagenolytic activity within the post-treatment strategy, we exclusively employed fluorescent gelatine as the substrate. In Fig. 8C, fluorescence was measured at three time points 2 h, 7 h and 24 h at the post-treatment strategy: the higher the fluorescence the greater the gelatinase activity. Only CHX (positive control for collagenase inhibition) exhibited a significant reduction in gelatinase activity within the biofilms. At 2 h, no significant differences were observed between the no treatment group and the other groups. However, after 7 h and 24 h, a significant higher gelatinase inhibition was found at the CHX group when compared to no treatment group (*p* = 0.0293 and *p* = 0.0246, respectively).

### Confirmation of root caries lesions development in the pre-treatment strategy

Visual inspection of the slabs revealed root caries-like lesions, evident through alterations in the colour of the dentine surface. Additionally, SEM images depicted deposits or sediments, some exhibiting filamentous characteristics, on the surfaces of groups pre-treated with anacardic acid LDT11 and cranberry (Supplementary Fig. 4). The µCT scanning of the dentine slabs, conducted before and after biofilm formation and cariogenic challenge, revealed visibly reduced mineral loss in all dentine slabs exposed to biofilms as a consequence from a successful dysbiosis-driven model. However, the group not exposed to sucrose cycles (G2 −) presented lower mineral loss than the group exposed to sucrose cycles without pre-treatments (G2 +). Slabs pre-treated with both anacardic acid LDT11 and cranberry seem to present lower mineral loss compared to the control G2 + . Dentine slabs maintained in the experimental condition without biofilm formation (no saliva inoculum) exhibited no mineral loss (G1) (Supplementary Fig. 5).

## Discussion

The objective of this research was to develop an in vitro cariogenic biofilm model and explore microbial modulation approaches for managing root caries. The presence of taxa previously found in natural root caries lesions (Fig. 3) such as *Lactobacillus*, *Streptococcus*, *Bifidobacterium*, *Prevotella*, *Selenomonas*, *Veillonella*, *Actinomyces*, *Treponema* and others confirms that we achieved the root caries biofilm engineering in vitro [5, 6, 26]. Two strategies were used to test substances that could modulate oral biofilms due to their bio-multifunctionality. In the first strategy, the tested compounds were incorporated before growing the biofilm (“pre-treatment strategy”). In the second strategy, mature biofilms were grown and then exposed to the compounds (“post-treatment strategy”). The advantages of the “post-treatment strategy” lie in its role as a viable substitute for artificial mouths, particularly in smaller oral microbiology laboratories, enabling rapid testing of various compounds, concentrations, and exposure times. However, a higher significance of the “pre-treatment strategy” to modulate the biofilms was observed, that can be attributed to its impact on biofilm formation, confirmed by the much less co-expression with the respective control in the metatranscriptome analysis than for the post-treated biofilms. Additionally, adding natural compounds to mature biofilms are less effective due to the biofilms’ resilient nature. While previous studies have explored microbiome modulation through surface pre-treatment or coating [27, 28], our focus is distinct, as we specifically target dentine collagen breakdown modulation to prevent/delay cavitation. Future clinical implications of these findings involve more feasibility to develop varnish products for application after professional tooth prophylaxis, as opposed to using mouthwashes, to implement substances for a dysbiosis reversal strategy in real-life scenarios.

### Complex biofilms development and root caries experimental model

Saliva collection is a non-invasive and simple method, which should be regarded as an important advantage as ex vivo inoculum for in vitro biofilm engineering. The use of saliva as inoculum to grow microcosm biofilm in vitro has been reported as sufficient to mimic the complexity of the oral microbiome [29]. Although it is known that the microbial composition in dental biofilms is quite different from that of saliva, it is described in the literature that the initial inoculum is less important than the media chosen to form the microcosm biofilm [21, 29]. Microcosm biofilms are in vitro version of natural biofilms and have been explored as a microbiota model due to their ease of manipulation and control. This method allows us to create a rich and diverse microbiota needed to develop the microcosm biofilm from the initial saliva, which can be used to artificially develop caries lesions in the laboratory [24]. From a ‘defined inoculum’ enriched with a very nutritive media, a complex biofilm reflecting the complexity of the mouth was created. Usually, saliva donors are caries-free individuals presenting good oral health. Here, we decided to include those with either a history of caries, periodontitis or gingivitis, due to the fact that individuals with inactive oral diseases carry a more similar microbial composition to the ones with active diseases than the disease-free [15, 17]. However, it is interesting to note that the composition of the metabolically active microbiota formed on dentine slabs without pre-treatments was very similar in terms of diversity to the initial inoculum, except for a few species that were likely unculturable (Fig. 3). Also, we achieved the biofilm complexity with a relatively low number of saliva donors (*N* = 4).

To our knowledge, this is the first time that the presence of virus and archaeal phyla in microcosm oral biofilms was described, confirming the complexity attained and highlighting the model’s utility as a surrogate for clinical biofilms. Interestingly, Thaumarchaeota-associated reads were exclusively observed in the groups subjected to sucrose cycles. Their presence in natural dental caries biofilms has been demonstrated previously [30], possibly linked to their ability to produce ammonia (Supplementary file 2).

The microbial communities of the initial inoculum and the control group without sucrose or any treatments showed striking similarity. This indicates a consistent pattern of gene expression between the inoculum and the cultured biofilm without the addition of sucrose, affirming the suitability of the laboratory conditions for cultivating oral biofilms. We used the SHI media formulated by Tian et al. [20], that is better suited to sustain the initial inoculum composition compared to basal media or artificial saliva. SHI media can be described as a hybrid, encompassing elements of both, basal media and artificial saliva, while incorporating blood and acetylmuramic acid to enhance the growth of more demanding anaerobic microorganisms. This would be relevant in the context of a root caries model given the presence of numerous periodontal pathobionts within the microbiome, as we previously discussed. The ease of incorporating proteolytic bacteria in the CBD, particularly those with collagen-binding capabilities can be facilitated by the collagen coating applied before growing mature biofilm in the post-treatment strategy. Furthermore, SHI media has 0.5% of sucrose in its composition, meaning that all biofilms had a minimum amount of sucrose throughout the entire experiment, and the low pH and dysbiotic condition were achieved in the increment of this sucrose concentration.

In the pre-treatment analysis, the groups exposed to 1% sucrose cycles reached the critical pH for demineralisation of root dentine of approximately 5.8 to 6.0 (Fig. 2), resembling a dysbiotic favouring condition [31]. The massive increase of lactobacilli (Fig. 3), combined with the low pH and the overexpression of carbohydrate transporters and glucosyltransferases (Fig. 5), confirmed that we could drive the biofilms to dysbiosis in the laboratory. Furthermore, expression pattern of differential expression genes clustered according to the presence or absence of sucrose cycles, and the group not exposed to sucrose had higher expression in amino acid, lipid, nucleotide and coenzyme transport and metabolism (Figs. 4 and 5). These results confirming the importance of sucrose cycles to drive biofilms towards dysbiotic conditions can be interpreted according to Sheiham and James discussion about avoiding the term “multifactorial” to characterize dental caries as a disease [32]. While various clinical factors can impact mineral loss, our data supports the idea that caries dysbiotic biofilms can be achieved with a singular change in the fermentable sugar concentration and frequency. Though, this just emphasizes the pivotal role of fermentable sugars, even in root caries, in shifting the biofilm from a homeostatic to a dysbiotic state.

Our model was conducted at 37 °C under anaerobic conditions to mimic the cariogenic environment. Altering the environmental conditions to aerobic, for example, could simulate a less aggressive lesion. Biofilm fermentation, which is primarily responsible for caries lesion development, occurs under anaerobic conditions, breaking down sugars to produce acids like lactic acid. These acids are the strongest to demineralize the tooth surfaces, leading to caries lesions. In an aerobic environment, these bacteria shift their metabolism towards other pathways, as the presence of oxygen inhibits their ability to produce acids at the same rate.

A simpler model featuring only one or a few organisms would not accurately represent the complexity of the system. For example, recent findings from our team reveal that methanogen archaea are overexpressing genes associated with methanogenesis in caries-free biofilms compared to caries-active ones (unpublished data). This suggests their potential involvement in pH regulation within this environment. In our model, we observed the growth of archaea, viruses and other microorganisms. A previous study evaluated the impact of antiseptic treatment on in vitro oral biofilms using two models: a controllable 14-species community and a more representative biofilm using human tongue as inoculum. They found that both biofilms exhibited similar stress responses when exposed to CHX for 5 min every 24 h to simulate mouthwash use, characterized by rapid regrowth to initial bacterial concentrations. The researchers concluded that alternative treatments are needed to selectively target disease-associated bacteria in the biofilm without affecting commensal microorganisms. We believe our study presents such an alternative: our results demonstrate the potential of pre-treating teeth surfaces as an effective alternative to mouthwashes [33].

### Collagenase activity in root caries biofilms

Matrix metalloproteinases (MMPs) are connected with the dentinal collagen degradation during the organic phase of root caries development, yet microbial collagenases could also participate in the process of collagen breakdown [4, 34, 35]. Microbial collagenase and MMPs exhibit distinct characteristics, potentially resulting in different outcomes in the dentinal collagen matrix. Unlike mammalian collagenases, which cleave collagen at a single site, bacterial collagenase from *C. histolyticum* performs multiple cleavages [36–38].

While such microbial collagenases activity has not been demonstrated experimentally [39, 40], we propose that bacteria are capable of breaking down the dentine collagen matrix due to their gene superexpression in root caries biofilms when compared to sound root surface biofilms [41]. However, in our dysbiotic biofilm model of the pre-treatment strategy, the collagenase activity was very low (Fig. 8). When compared to the isolated *C. hystolyticum* collagenase, only the control samples without pre-treatment and without sugar cycles showed any significant collagenase activity. Without pre-treatment or sucrose cycles, the control samples surprisingly exhibited significant collagenase activity (less sugar = more proteolytic microbes and then more collagenases). Despite the notably low collagenase activity during pre-treatment, arguably the most crucial outcome derived from this experiment is the affirmation that microbial collagenases within biofilms from carious lesions indeed are capable of breaking down gelatine (Fig. 8B).

### Cranberry and anacardic acid are potential agents for modulating root caries biofilms

There is evidence on the multifunctionality of cranberry and its components, specifically proanthocyanidin. Cranberry’s antimicrobial effect against oral bacteria has been shown in planktonic cells [12], which we also showed here. The anti-proteolytic activity has been shown in proanthocyanidin-biomodified demineralized dentine matrix [13]. Cranberry proanthocyanidins anti-biofilm properties against *Pseudomonas aeruginosa* (disruption of preformed biofilms) [42] corroborates with our results on cells detaching in the mature biofilms. The pH of the media in the cranberry-pre-treated dentine slab group was lower on day 2 maybe it can explain the metatranscriptome dissimilarity. Also, it could explain the anti-biofilm characteristics observed in the post-treatment with cranberry. Next steps would involve the characterisation of the cranberry extracts to validate our data, using isolated components such as proanthoancin.

Furthermore, the presence of anacardic acid LDT11 resulted in no expression of the collagenase gene, and low expression in the presence of cranberry (Supplementary Fig. 3). Additionally, pre-treatment with anacardic acid LDT11 induced alterations in metal binding gene expression, potentially linked to its anti-enzymatic capacity (Fig. 4). Although we observed inhibition of collagenase gene expression, the enzyme activity was too low to detect inhibition by the compounds in our collagenase/gelatinase activity assay (Fig. 8). We believe that a longer incubation period might reveal more significant collagenase activity, suggesting that the model should be enhanced for this assay. Interestingly, cranberry showed potential for gelatinase inhibition, which warrants further exploration. Although CHX exhibited some gelatinase activity inhibition in the post-treatment strategy, due to its association with antimicrobial resistance, alternatives like cranberry may be more attractive clinically, although our results were only slightly significant.

Pre-treatment of root surfaces with LDT11 and cranberry has the potential to demonstrate a significant reduction in bacterial collagenase activity and enhanced modulation of dysbiotic biofilms compared to post-treatment application. Further investigation is needed regarding potential chelation with calcium in the experimental model in the presence of dentine specimens, as it may have constrained its effectiveness in this context. The deposits observed in the SEM can be suggestive of dentine biomodification by both anacardic acid and cranberry. This implies that the use of these compounds may also preserve the collagenolytic matrix in root tissues and in root caries management, which the trend was observed in the μ-CT analysis (Supplementary Fig. 5). Our data revealed a reduced area of mineral loss in the pre-treated samples, suggesting a potential decrease in lesion size. The next steps include further exploring this data on lesion size and testing the substances’ ability to promote cross-linking in dentine. Additionally, we have already synthesized a varnish containing the compounds for future testing.

### Limitations

This model has some limitations. Although it is a complex biofilm model, it is still an in vitro study. Bovine dentine was used as a surrogate for human dentine due to its convenience and suitability for studies involving the activity of MMPs [36]. For instance, bovine teeth are suitable for caries development, as they are more uniform in terms of mineral composition and easier to manipulate in laboratory due to their size [43]. One could argue that CHX should also be used as a control in the pre-treatment strategy. However, we opted to use sucrose without pre-treatment as the control because it provides a relevant baseline for comparison, representing the condition where no compounds are introduced prior to the experimental treatments. This approach allows us to evaluate the natural progression and biofilm formation in the absence of any intervention, thereby serving as an effective negative control. Additionally, our focus was on the efficacy of post-treatment interventions in biofilm modulation by altering the surface. Using CHX, a potent antimicrobial agent, as a pre-treatment could significantly alter the initial conditions by influencing biofilm formation, introducing additional variables that might confound the results. Moreover, it is conceivable that the substantial alteration observed in the biofilm gene expression on the pre-treated surfaces may be attributed to the biomodification of the dentine surface and this could explain the microbiome modulation. However, further investigation is required to elucidate this aspect.

## Conclusion

In conclusion, a root caries-like biofilm and lesions were successfully created, demonstrating the successful development of caries-specific dysbiosis in vitro. The metatranscriptome analysis revealed a more pronounced effect when substances were used as a pre-treatment of dentine before the biofilm development and cariogenic challenges. These substances effectively reduced lesion sizes and delayed gelatine hydrolysis. Further research needs to be carried out to improve microbial control and modulation in order to maintain dental tooth health, and the current challenges are to reverse this dysbiosis in vitro and explore alternative methods for isolating microbial collagenolytic function in a laboratory setting. By doing so, we can test a broader range of substances and develop innovative treatments for root caries.

### Supplementary Information

Additional file 1: Supplementary table 1. Summary of the pre-treatment strategy groups. Supplementary table 2. Summary of the statistical analysis for the pH monitoring in Figure 2. Supplementary table 3. Statistical analysis summary for the antimicrobial activity post-treatment strategy in Figure 6D. Supplementary table 4. Statistical analysis summary for the antimicrobial activity post-treatment strategy in Figure 6E. Supplementary Figure 1. Cluster analysis of differential expression genes. Hierarchical clustering analysis was carried out with the log10(Fragments Per Kilobase of transcript per Million mapped reads=FPKM+1) of union differential expression genes of all comparison groups under different experimental conditions. Genes in one cluster have similar expression levels. The x axis represents sample names, the y axis represents the corrected expression level value. Supplementary Figure 2 (A=Biological processes, B=Cellular components, C=Metabolic functions). TopGO DAG (Directed Acyclic Graph, DAG) of the enriched GO (Gene Ontology) term of differential expression genes and its hierarchical relation for the Control 2 (G2+) and Control 1 (G2-). Each node represents a GO term, and Top 10 GO terms are boxed. The darker the color is, the higher is the enrichment level of the term. The name and p-value of each term are present on the node. Supplementary Figure 3. Functional annotation with EggNOG (Evolutionary Genealogy of Genes: Non-supervised Orthologous Groups) – unigene total absolute of the Collagenases U32 gene expression. Supplementary Figure 4. SEM images from dentine slabs after exposure to dysbiotic root caries-like biofilms and control (Control - = “G2-“, Control+sugar = “G2+”).Supplementary Figure 5. Examples of micro computed tomography (μ-CT) of dentine slabs confirming the presence of root caries-like lesions and mineral loss (blue areas represents the difference in the reference and target images, I.e., the demineralised area). A) 2D images for the groups exposed or not to sucrose cycles; B) 3D images for the following groups: G1; without inoculum; G2+; with inoculum, no pre-treatment, with sucrose cycles; G2- ; with inoculum, no pre-treatment, no sucrose cycles; G3; with inoculum, with LDT11 pre-treatment, with sucrose cycles; G4; with inoculum, with cranberry pre-treatment, with sucrose cycles.

Additional file 2. Taxonomic analysis, unigene relative abundance table.

## Data Availability

PRJNA1083328.
