# Supplementary material for: Engineering a dysbiotic biofilm model for testing root caries interventions through microbial modulation
Source: Microbiome. 2024 Aug 6;12:145. doi: 10.1186/s40168-024-01862-5 (PMC11302357; doi:10.1186/s40168-024-01862-5)
Supplement: Supplementary file 2 — Additional file 1: Supplementary table 1. Summary of the pre-treatment strategy groups. Supplementary table 2. Summary of the statistical analysis for the pH monitoring in Figure 2. Supplementary table 3. Statistical analysis summary for the antimicrobial activity post-treatment strategy in Figure 6D. Supplementary table 4. Statistical analysis summary for the antimicrobial activity post-treatment strategy in Figure 6E. Supplementary Figure 1. Cluster analysis of differential expression genes. Hierarchical clustering analysis was carried out with the log10(Fragments Per Kilobase of transcript per Million mapped reads=FPKM+1) of union differential expression genes of all comparison groups under different experimental conditions. Genes in one cluster have similar expression levels. The x axis represents sample names, the y axis represents the corrected expression level value. Supplementary Figure 2 (A=Biological processes, B=Cellular components, C=Metabolic functions). TopGO DAG (Directed Acyclic Graph, DAG) of the enriched GO (Gene Ontology) term of differential expression genes and its hierarchical relation for the Control 2 (G2+) and Control 1 (G2-). Each node represents a GO term, and Top 10 GO terms are boxed. The darker the color is, the higher is the enrichment level of the term. The name and p-value of each term are present on the node. Supplementary Figure 3. Functional annotation with EggNOG (Evolutionary Genealogy of Genes: Non-supervised Orthologous Groups) – unigene total absolute of the Collagenases U32 gene expression. Supplementary Figure 4. SEM images from dentine slabs after exposure to dysbiotic root caries-like biofilms and control (Control - = “G2-“, Control+sugar = “G2+”).Supplementary Figure 5. Examples of micro computed tomography (μ-CT) of dentine slabs confirming the presence of root caries-like lesions and mineral loss (blue areas represents the difference in the reference and target images, I.e., the demineralised area). A) 2D images for th [file 40168_2024_1862_MOESM1_ESM.docx]

**Engineering a dysbiotic biofilm model for testing root caries interventions through microbial modulation**

N **Dame‐Teixeira**, R **El‐Gendy**, AS **Oliveira,** CA **Holanda,** LAS **Romeiro,** T **Do**

SUPPLEMENTARY MATERIAL

**Supplementary table 1.** Summary of the pre-treatment strategy groups.

| **Type of intervention**  **(group)** | **Saliva inoculum** | **Demineralization Sucrose cycle** | **Demineralisation High pH** | **Pre-treatment with LDT11** | **Pre-treatment with Cranberry** |
| --- | --- | --- | --- | --- | --- |
| Group 1 (G1) | - | - | - | - | - |
| G2+ | + | + | - | - | - |
| G2- | + | - | + | - | - |
| G3 | + | + | - | + | - |
| G4 | + | + | - | - | + |

**Supplementary table 2.** Summary of the statistical analysis for the pH monitoring in Figure 2.

| \| **2way ANOVA, multiple comparisons – 10am before adding sugar – Pre-treatment**  **Within each row, compare columns (simple effects within rows)** \| \| \| \| \| \|  \|  \|  \| \| --- \| --- \| --- \| --- \| --- \| --- \| --- \| --- \| --- \| \| Tukey's multiple comparisons test \| Mean Diff. \| 95.00% CI of diff. \| Below threshold? \| Summary \| Adjusted P Value \|  \|  \|  \| \| Control No treatment \|  \|  \|  \|  \|  \|  \|  \|  \| \| Day 2 vs. Day 4 \| -0.6850 \| -3.485 to 2.115 \| No \| ns \| 0.2164 \|  \|  \|  \| \| Day 2 vs. Day 6 \| -0.8550 \| -4.142 to 2.432 \| No \| ns \| 0.2039 \|  \|  \|  \| \| Day 2 vs. Day 8 \| -0.7200 \| -3.399 to 1.959 \| No \| ns \| 0.1975 \|  \|  \|  \| \| Day 2 vs. Day 10 \| -0.8450 \| -4.132 to 2.442 \| No \| ns \| 0.2063 \|  \|  \|  \| \| Day 4 vs. Day 6 \| -0.1700 \| -0.6570 to 0.3170 \| No \| ns \| 0.1531 \|  \|  \|  \| \| Day 4 vs. Day 8 \| -0.03500 \| -0.1568 to 0.08676 \| No \| ns \| 0.1850 \|  \|  \|  \| \| Day 4 vs. Day 10 \| -0.1600 \| -0.6470 to 0.3270 \| No \| ns \| 0.1624 \|  \|  \|  \| \| Day 6 vs. Day 8 \| 0.1350 \| -0.4738 to 0.7438 \| No \| ns \| 0.2378 \|  \|  \|  \| \| Day 6 vs. Day 10 \| 0.01000 \| 0.01000 to 0.01000 \| Yes \| **** \| <0.0001 \|  \|  \|  \| \| Day 8 vs. Day 10 \| -0.1250 \| -0.7338 to 0.4838 \| No \| ns \| 0.2559 \|  \|  \|  \| \|  \|  \|  \|  \|  \|  \|  \|  \|  \| \| LDT11 100 μg/mL \|  \|  \|  \|  \|  \|  \|  \|  \| \| Day 2 vs. Day 4 \| -0.6150 \| -2.685 to 1.455 \| No \| ns \| 0.1792 \|  \|  \|  \| \| Day 2 vs. Day 6 \| -0.7300 \| -2.435 to 0.9746 \| No \| ns \| 0.1252 \|  \|  \|  \| \| Day 2 vs. Day 8 \| -0.6650 \| -3.222 to 1.892 \| No \| ns \| 0.2039 \|  \|  \|  \| \| Day 2 vs. Day 10 \| -0.7550 \| -3.068 to 1.558 \| No \| ns \| 0.1635 \|  \|  \|  \| \| Day 4 vs. Day 6 \| -0.1150 \| -0.4803 to 0.2503 \| No \| ns \| 0.1693 \|  \|  \|  \| \| Day 4 vs. Day 8 \| -0.05000 \| -0.5370 to 0.4370 \| No \| ns \| 0.4768 \|  \|  \|  \| \| Day 4 vs. Day 10 \| -0.1400 \| -0.3835 to 0.1035 \| No \| ns \| 0.0940 \|  \|  \|  \| \| Day 6 vs. Day 8 \| 0.06500 \| -0.7873 to 0.9173 \| No \| ns \| 0.5995 \|  \|  \|  \| \| Day 6 vs. Day 10 \| -0.02500 \| -0.6338 to 0.5838 \| No \| ns \| 0.8430 \|  \|  \|  \| \| Day 8 vs. Day 10 \| -0.09000 \| -0.3335 to 0.1535 \| No \| ns \| 0.1447 \|  \|  \|  \| \|  \|  \|  \|  \|  \|  \|  \|  \|  \| \| Cranberry 100 μg/mL \|  \|  \|  \|  \|  \|  \|  \|  \| \| Day 2 vs. Day 4 \| -0.8750 \| -1.727 to -0.02269 \| Yes \| * \| 0.0479 \|  \|  \|  \| \| Day 2 vs. Day 6 \| -1.000 \| -1.244 to -0.7565 \| Yes \| * \| 0.0211 \|  \|  \|  \| \| Day 2 vs. Day 8 \| -0.8900 \| -0.8900 to -0.8900 \| Yes \| **** \| <0.0001 \|  \|  \|  \| \| Day 2 vs. Day 10 \| -1.050 \| -1.294 to -0.8065 \| Yes \| * \| 0.0211 \|  \|  \|  \| \| Day 4 vs. Day 6 \| -0.1250 \| -0.7338 to 0.4838 \| No \| ns \| 0.2559 \|  \|  \|  \| \| Day 4 vs. Day 8 \| -0.01500 \| -0.8673 to 0.8373 \| No \| ns \| 0.9843 \|  \|  \|  \| \| Day 4 vs. Day 10 \| -0.1750 \| -1.271 to 0.9208 \| No \| ns \| 0.3237 \|  \|  \|  \| \| Day 6 vs. Day 8 \| 0.1100 \| -0.1335 to 0.3535 \| No \| ns \| 0.1188 \|  \|  \|  \| \| Day 6 vs. Day 10 \| -0.05000 \| -0.5370 to 0.4370 \| No \| ns \| 0.4768 \|  \|  \|  \| \| Day 8 vs. Day 10 \| -0.1600 \| -0.4035 to 0.08352 \| No \| ns \| 0.0827 \|  \|  \|  \| \|  \|  \|  \|  \|  \|  \|  \|  \|  \| |  |  |  |  |  |  |  |  |
| --- | --- | --- | --- | --- | --- | --- | --- | --- | --- | --- | --- | --- | --- | --- | --- | --- | --- | --- | --- | --- | --- | --- | --- | --- | --- | --- | --- | --- | --- | --- | --- | --- | --- | --- | --- | --- | --- | --- | --- | --- | --- | --- | --- | --- | --- | --- | --- | --- | --- | --- | --- | --- | --- | --- | --- | --- | --- | --- | --- | --- | --- | --- | --- | --- | --- | --- | --- | --- | --- | --- | --- | --- | --- | --- | --- | --- | --- | --- | --- | --- | --- | --- | --- | --- | --- | --- | --- | --- | --- | --- | --- | --- | --- | --- | --- | --- | --- | --- | --- | --- | --- | --- | --- | --- | --- | --- | --- | --- | --- | --- | --- | --- | --- | --- | --- | --- | --- | --- | --- | --- | --- | --- | --- | --- | --- | --- | --- | --- | --- | --- | --- | --- | --- | --- | --- | --- | --- | --- | --- | --- | --- | --- | --- | --- | --- | --- | --- | --- | --- | --- | --- | --- | --- | --- | --- | --- | --- | --- | --- | --- | --- | --- | --- | --- | --- | --- | --- | --- | --- | --- | --- | --- | --- | --- | --- | --- | --- | --- | --- | --- | --- | --- | --- | --- | --- | --- | --- | --- | --- | --- | --- | --- | --- | --- | --- | --- | --- | --- | --- | --- | --- | --- | --- | --- | --- | --- | --- | --- | --- | --- | --- | --- | --- | --- | --- | --- | --- | --- | --- | --- | --- | --- | --- | --- | --- | --- | --- | --- | --- | --- | --- | --- | --- | --- | --- | --- | --- | --- | --- | --- | --- | --- | --- | --- | --- | --- | --- | --- | --- | --- | --- | --- | --- | --- | --- | --- | --- | --- | --- | --- | --- | --- | --- | --- | --- | --- | --- | --- | --- | --- | --- | --- | --- | --- | --- | --- | --- | --- | --- | --- | --- | --- | --- | --- | --- | --- | --- | --- | --- | --- | --- | --- | --- | --- | --- | --- | --- | --- | --- | --- | --- | --- | --- | --- | --- | --- | --- | --- | --- | --- | --- | --- | --- | --- | --- | --- | --- | --- | --- | --- | --- | --- | --- | --- | --- | --- | --- | --- | --- | --- | --- | --- | --- | --- | --- | --- | --- | --- | --- | --- | --- | --- | --- | --- | --- | --- | --- | --- | --- | --- |
| **2way ANOVA, multiple comparisons – 4pm after adding sugar – pre-treatment**  **Within each row, compare columns (simple effects within rows)**  **Group C = "No treatment (with sucrose cycles)"**   \| Dunnett's multiple comparisons test \| Mean Diff. \| 95.00% CI of diff. \| Below threshold? \| Summary \| Adjusted P Value \|  \|  \|  \| \| --- \| --- \| --- \| --- \| --- \| --- \| --- \| --- \| --- \| \|  \|  \|  \|  \|  \|  \|  \|  \|  \| \| Day 1 \|  \|  \|  \|  \|  \|  \|  \|  \| \| Group C vs. Negative control (no biofilm) \| -0.8600 \| -2.243 to 0.5228 \| No \| ns \| >0.9999 \|  \|  \|  \| \| Group C vs. No treatment (no sucrose cycles) \| 0.07000 \| -0.5913 to 0.7313 \| No \| ns \| 0.8783 \|  \|  \|  \| \| Group C vs. LDT11 100 μg/mL \| -0.08500 \| -1.083 to 0.9133 \| No \| ns \| 0.6664 \|  \|  \|  \| \| Group C vs. Cranberry 100 μg/mL \| -0.1200 \| -1.503 to 1.263 \| No \| ns \| >0.9999 \|  \|  \|  \| \|  \|  \|  \|  \|  \|  \|  \|  \|  \| \| Day 2 \|  \|  \|  \|  \|  \|  \|  \|  \| \| Group C vs. Negative control (no biofilm) \| -1.885 \| -2.302 to -1.468 \| Yes \| ** \| 0.0037 \|  \|  \|  \| \| Group C vs. No treatment (no sucrose cycles) \| -0.6200 \| -1.060 to -0.1804 \| Yes \| * \| 0.0329 \|  \|  \|  \| \| Group C vs. LDT11 100 μg/mL \| -0.09500 \| -0.6765 to 0.4865 \| No \| ns \| >0.9999 \|  \|  \|  \| \| Group C vs. Cranberry 100 μg/mL \| 0.05000 \| -0.3896 to 0.4896 \| No \| ns \| 0.5578 \|  \|  \|  \| \|  \|  \|  \|  \|  \|  \|  \|  \|  \| \| Day 4 \|  \|  \|  \|  \|  \|  \|  \|  \| \| Group C vs. Negative control (no biofilm) \| -1.545 \| -1.654 to -1.436 \| Yes \| ** \| 0.0046 \|  \|  \|  \| \| Group C vs. No treatment (no sucrose cycles) \| -1.250 \| -1.687 to -0.8128 \| Yes \| * \| 0.0176 \|  \|  \|  \| \| Group C vs. LDT11 100 μg/mL \| -0.05500 \| -0.1643 to 0.05429 \| No \| ns \| 0.0990 \|  \|  \|  \| \| Group C vs. Cranberry 100 μg/mL \| -0.05000 \| -0.7057 to 0.6057 \| No \| ns \| 0.5618 \|  \|  \|  \| \|  \|  \|  \|  \|  \|  \|  \|  \|  \| \| Day 6 \|  \|  \|  \|  \|  \|  \|  \|  \| \| Group C vs. Negative control (no biofilm) \| -1.570 \| -2.276 to -0.8637 \| Yes \| * \| 0.0108 \|  \|  \|  \| \| Group C vs. No treatment (no sucrose cycles) \| -1.220 \| -2.582 to 0.1419 \| No \| ns \| >0.9999 \|  \|  \|  \| \| Group C vs. LDT11 100 μg/mL \| 0.1150 \| -1.391 to 1.621 \| No \| ns \| >0.9999 \|  \|  \|  \| \| Group C vs. Cranberry 100 μg/mL \| 0.05500 \| -1.451 to 1.561 \| No \| ns \| >0.9999 \|  \|  \|  \| \|  \|  \|  \|  \|  \|  \|  \|  \|  \| \| Day 8 \|  \|  \|  \|  \|  \|  \|  \|  \| \| Group C vs. Negative control (no biofilm) \| -1.630 \| -1.851 to -1.409 \| Yes \| ** \| 0.0027 \|  \|  \|  \| \| Group C vs. No treatment (no sucrose cycles) \| -1.280 \| -1.464 to -1.096 \| Yes \| ** \| 0.0011 \|  \|  \|  \| \| Group C vs. LDT11 100 μg/mL \| 0.03000 \| -0.1912 to 0.2512 \| No \| ns \| 0.6156 \|  \|  \|  \| \| Group C vs. Cranberry 100 μg/mL \| -0.01000 \| -0.2312 to 0.2112 \| No \| ns \| 0.9634 \|  \|  \|  \| \|  \|  \|  \|  \|  \|  \|  \|  \|  \| \| Day 10 \|  \|  \|  \|  \|  \|  \|  \|  \| \| Group C vs. Negative control (no biofilm) \| -1.570 \| -2.226 to -0.9143 \| Yes \| * \| 0.0209 \|  \|  \|  \| \| Group C vs. No treatment (no sucrose cycles) \| -1.385 \| -1.717 to -1.053 \| Yes \| ** \| 0.0062 \|  \|  \|  \| \| Group C vs. LDT11 100 μg/mL \| -0.01500 \| -0.5965 to 0.5665 \| No \| ns \| >0.9999 \|  \|  \|  \| \| Group C vs. Cranberry 100 μg/mL \| 0.05500 \| -0.2768 to 0.3868 \| No \| ns \| 0.5166 \|  \|  \|  \| |  |  |  |  |  |  |  |  |

| **Mixed effect analysis with multiple comparisons, Post-treatment**  **Within each column, compare rows (simple effects within columns)** | | |  |  |  |  |  |  |
| --- | --- | --- | --- | --- | --- | --- | --- | --- |
| Tukey's multiple comparisons test | Predicted (LS) mean diff. | 95.00% CI of diff. | Below threshold? | Summary | Adjusted P Value |  |  |  |
| Saliva |  |  |  |  |  |  |  |  |
| Day 1 vs. Day 2 | 0.2625 | -0.08181 to 0.6068 | No | ns | 0.2079 |  |  |  |
| Day 1 vs. Day 3 | -0.2273 | -0.5583 to 0.1037 | No | ns | 0.3046 |  |  |  |
| Day 1 vs. Day 4 | -0.2953 | -0.6263 to 0.03568 | No | ns | 0.0989 |  |  |  |
| Day 1 vs. Day 5 | -0.5213 | -0.8523 to -0.1903 | Yes | *** | 0.0008 |  |  |  |
| Day 1 vs. Day 6 | -0.6853 | -1.016 to -0.3543 | Yes | **** | <0.0001 |  |  |  |
| Day 2 vs. Day 3 | -0.4898 | -0.8208 to -0.1588 | Yes | ** | 0.0017 |  |  |  |
| Day 2 vs. Day 4 | -0.5578 | -0.8888 to -0.2268 | Yes | *** | 0.0004 |  |  |  |
| Day 2 vs. Day 5 | -0.7838 | -1.115 to -0.4528 | Yes | **** | <0.0001 |  |  |  |
| Day 2 vs. Day 6 | -0.9478 | -1.279 to -0.6168 | Yes | **** | <0.0001 |  |  |  |
| Day 3 vs. Day 4 | -0.06800 | -0.3760 to 0.2400 | No | ns | 0.9815 |  |  |  |
| Day 3 vs. Day 5 | -0.2940 | -0.6020 to 0.01396 | No | ns | 0.0670 |  |  |  |
| Day 3 vs. Day 6 | -0.4580 | -0.7660 to -0.1500 | Yes | ** | 0.0016 |  |  |  |
| Day 4 vs. Day 5 | -0.2260 | -0.5340 to 0.08196 | No | ns | 0.2413 |  |  |  |
| Day 4 vs. Day 6 | -0.3900 | -0.6980 to -0.08204 | Yes | ** | 0.0079 |  |  |  |
| Day 5 vs. Day 6 | -0.1640 | -0.4720 to 0.1440 | No | ns | 0.5710 |  |  |  |
|  |  |  |  |  |  |  |  |  |
| No saliva |  |  |  |  |  |  |  |  |
| Day 1 vs. Day 2 | 0.4725 | -0.1429 to 1.088 | No | ns | 0.2020 |  |  |  |
| Day 1 vs. Day 3 | 0.4475 | -0.1679 to 1.063 | No | ns | 0.2496 |  |  |  |
| Day 1 vs. Day 4 | 0.3525 | -0.2629 to 0.9678 | No | ns | 0.4951 |  |  |  |
| Day 1 vs. Day 5 | 0.2175 | -0.3979 to 0.8328 | No | ns | 0.8757 |  |  |  |
| Day 1 vs. Day 6 | 0.2675 | -0.3479 to 0.8828 | No | ns | 0.7525 |  |  |  |
| Day 2 vs. Day 3 | -0.02500 | -0.5119 to 0.4619 | No | ns | >0.9999 |  |  |  |
| Day 2 vs. Day 4 | -0.1200 | -0.6069 to 0.3669 | No | ns | 0.9702 |  |  |  |
| Day 2 vs. Day 5 | -0.2550 | -0.7419 to 0.2319 | No | ns | 0.5878 |  |  |  |
| Day 2 vs. Day 6 | -0.2050 | -0.6919 to 0.2819 | No | ns | 0.7758 |  |  |  |
| Day 3 vs. Day 4 | -0.09500 | -0.5819 to 0.3919 | No | ns | 0.9893 |  |  |  |
| Day 3 vs. Day 5 | -0.2300 | -0.7169 to 0.2569 | No | ns | 0.6849 |  |  |  |
| Day 3 vs. Day 6 | -0.1800 | -0.6669 to 0.3069 | No | ns | 0.8543 |  |  |  |
| Day 4 vs. Day 5 | -0.1350 | -0.6219 to 0.3519 | No | ns | 0.9512 |  |  |  |
| Day 4 vs. Day 6 | -0.08500 | -0.5719 to 0.4019 | No | ns | 0.9936 |  |  |  |
| Day 5 vs. Day 6 | 0.05000 | -0.4369 to 0.5369 | No | ns | 0.9995 |  |  |  |
|  |  |  |  |  |  |  |  |  |
|  |  |  |  |  |  |  |  |  |

**Supplementary table 3.** Statistical analysis summary for the antimicrobial activity post-treatment strategy in Figure 6D.

| 2way ANOVA with multiple comparisons  Within each row, compare columns (simple effects within rows) | | | |  |  |
| --- | --- | --- | --- | --- | --- |
| Tukey's multiple comparisons test | Predicted (LS) mean diff. | 95.00% CI of diff. | Below threshold? | Summary | Adjusted P Value |
|  |  |  |  |  |  |
| 10 min |  |  |  |  |  |
| No treatment vs. LDT11 50 μg/mL | -0.6941 | -2.045 to 0.6571 | No | ns | 0.6497 |
| No treatment vs. LDT11 100 μg/mL | -0.2803 | -1.705 to 1.144 | No | ns | 0.9916 |
| No treatment vs. Chlorexidine 100 μg/mL | 1.762 | 0.3373 to 3.186 | Yes | ** | 0.0076 |
| No treatment vs. Cranberry 50 μg/mL | 1.433 | 0.008342 to 2.857 | Yes | * | 0.0479 |
| No treatment vs. Cranberry 100 μg/mL | 1.011 | -0.4139 to 2.435 | No | ns | 0.3013 |
| LDT11 50 μg/mL vs. LDT11 100 μg/mL | 0.4138 | -0.7779 to 1.606 | No | ns | 0.9049 |
| LDT11 50 μg/mL vs. Chlorexidine 100 μg/mL | 2.456 | 1.264 to 3.647 | Yes | **** | <0.0001 |
| LDT11 50 μg/mL vs. Cranberry 50 μg/mL | 2.127 | 0.9351 to 3.319 | Yes | **** | <0.0001 |
| LDT11 50 μg/mL vs. Cranberry 100 μg/mL | 1.705 | 0.5129 to 2.896 | Yes | ** | 0.0013 |
| LDT11 100 μg/mL vs. Chlorexidine 100 μg/mL | 2.042 | 0.7680 to 3.316 | Yes | *** | 0.0003 |
| LDT11 100 μg/mL vs. Cranberry 50 μg/mL | 1.713 | 0.4390 to 2.987 | Yes | ** | 0.0029 |
| LDT11 100 μg/mL vs. Cranberry 100 μg/mL | 1.291 | 0.01684 to 2.565 | Yes | * | 0.0454 |
| Chlorexidine 100 μg/mL vs. Cranberry 50 μg/mL | -0.3289 | -1.603 to 0.9451 | No | ns | 0.9717 |
| Chlorexidine 100 μg/mL vs. Cranberry 100 μg/mL | -0.7511 | -2.025 to 0.5228 | No | ns | 0.5060 |
| Cranberry 50 μg/mL vs. Cranberry 100 μg/mL | -0.4222 | -1.696 to 0.8518 | No | ns | 0.9206 |
|  |  |  |  |  |  |
| 30 min |  |  |  |  |  |
| No treatment vs. LDT11 50 μg/mL | 0.01134 | -0.8227 to 0.8454 | No | ns | >0.9999 |
| No treatment vs. LDT11 100 μg/mL | 0.03786 | -0.7962 to 0.8719 | No | ns | >0.9999 |
| No treatment vs. Chlorexidine 100 μg/mL | -0.1052 | -0.9733 to 0.7628 | No | ns | 0.9992 |
| No treatment vs. Cranberry 50 μg/mL | -0.08900 | -0.9230 to 0.7450 | No | ns | 0.9995 |
| No treatment vs. Cranberry 100 μg/mL | -0.1920 | -1.026 to 0.6420 | No | ns | 0.9829 |
| LDT11 50 μg/mL vs. LDT11 100 μg/mL | 0.02652 | -0.8075 to 0.8605 | No | ns | >0.9999 |
| LDT11 50 μg/mL vs. Chlorexidine 100 μg/mL | -0.1166 | -0.9847 to 0.7515 | No | ns | 0.9986 |
| LDT11 50 μg/mL vs. Cranberry 50 μg/mL | -0.1003 | -0.9344 to 0.7337 | No | ns | 0.9992 |
| LDT11 50 μg/mL vs. Cranberry 100 μg/mL | -0.2034 | -1.037 to 0.6307 | No | ns | 0.9780 |
| LDT11 100 μg/mL vs. Chlorexidine 100 μg/mL | -0.1431 | -1.011 to 0.7250 | No | ns | 0.9963 |
| LDT11 100 μg/mL vs. Cranberry 50 μg/mL | -0.1269 | -0.9609 to 0.7072 | No | ns | 0.9975 |
| LDT11 100 μg/mL vs. Cranberry 100 μg/mL | -0.2299 | -1.064 to 0.6041 | No | ns | 0.9626 |
| Chlorexidine 100 μg/mL vs. Cranberry 50 μg/mL | 0.01624 | -0.8518 to 0.8843 | No | ns | >0.9999 |
| Chlorexidine 100 μg/mL vs. Cranberry 100 μg/mL | -0.08679 | -0.9549 to 0.7813 | No | ns | 0.9997 |
| Cranberry 50 μg/mL vs. Cranberry 100 μg/mL | -0.1030 | -0.9371 to 0.7310 | No | ns | 0.9991 |

**Supplementary table 4.** Statistical analysis summary for the antimicrobial activity post-treatment strategy in Figure 6E.

| Within each row, compare columns (simple effects within rows) |  |  |  |  |  |
| --- | --- | --- | --- | --- | --- |
| Tukey's multiple comparisons test | Mean Diff. | 95.00% CI of diff. | Below threshold? | Summary | Adjusted P Value |
| Total aerobes |  |  |  |  |  |
| No treatment vs. LDT11 50 | -0.1000 | -0.5177 to 0.3177 | No | ns | 0.9782 |
| No treatment vs. LDT11 100 | 0.06575 | -0.3520 to 0.4835 | No | ns | 0.9968 |
| No treatment vs. CHX | 0.8980 | 0.4803 to 1.316 | Yes | **** | <0.0001 |
| No treatment vs. Cran 50 | -0.2043 | -0.6220 to 0.2135 | No | ns | 0.6841 |
| No treatment vs. Cran 100 | -0.06950 | -0.4872 to 0.3482 | No | ns | 0.9958 |
| LDT11 50 vs. LDT11 100 | 0.1657 | -0.2520 to 0.5835 | No | ns | 0.8369 |
| LDT11 50 vs. CHX | 0.9980 | 0.5803 to 1.416 | Yes | **** | <0.0001 |
| LDT11 50 vs. Cran 50 | -0.1043 | -0.5220 to 0.3135 | No | ns | 0.9738 |
| LDT11 50 vs. Cran 100 | 0.03050 | -0.3872 to 0.4482 | No | ns | >0.9999 |
| LDT11 100 vs. CHX | 0.8323 | 0.4145 to 1.250 | Yes | **** | <0.0001 |
| LDT11 100 vs. Cran 50 | -0.2700 | -0.6877 to 0.1477 | No | ns | 0.3929 |
| LDT11 100 vs. Cran 100 | -0.1352 | -0.5530 to 0.2825 | No | ns | 0.9231 |
| CHX vs. Cran 50 | -1.102 | -1.520 to -0.6845 | Yes | **** | <0.0001 |
| CHX vs. Cran 100 | -0.9675 | -1.385 to -0.5498 | Yes | **** | <0.0001 |
| Cran 50 vs. Cran 100 | 0.1348 | -0.2830 to 0.5525 | No | ns | 0.9243 |
|  |  |  |  |  |  |
| Total anaerobes |  |  |  |  |  |
| No treatment vs. LDT11 50 | 0.05175 | -0.3660 to 0.4695 | No | ns | 0.9990 |
| No treatment vs. LDT11 100 | 0.1403 | -0.2775 to 0.5580 | No | ns | 0.9115 |
| No treatment vs. CHX | 1.126 | 0.7083 to 1.544 | Yes | **** | <0.0001 |
| No treatment vs. Cran 50 | 0.09900 | -0.3187 to 0.5167 | No | ns | 0.9791 |
| No treatment vs. Cran 100 | 0.2757 | -0.1420 to 0.6935 | No | ns | 0.3698 |
| LDT11 50 vs. LDT11 100 | 0.08850 | -0.3292 to 0.5062 | No | ns | 0.9873 |
| LDT11 50 vs. CHX | 1.074 | 0.6565 to 1.492 | Yes | **** | <0.0001 |
| LDT11 50 vs. Cran 50 | 0.04725 | -0.3705 to 0.4650 | No | ns | 0.9993 |
| LDT11 50 vs. Cran 100 | 0.2240 | -0.1937 to 0.6417 | No | ns | 0.5954 |
| LDT11 100 vs. CHX | 0.9857 | 0.5680 to 1.403 | Yes | **** | <0.0001 |
| LDT11 100 vs. Cran 50 | -0.04125 | -0.4590 to 0.3765 | No | ns | 0.9997 |
| LDT11 100 vs. Cran 100 | 0.1355 | -0.2822 to 0.5532 | No | ns | 0.9226 |
| CHX vs. Cran 50 | -1.027 | -1.445 to -0.6093 | Yes | **** | <0.0001 |
| CHX vs. Cran 100 | -0.8503 | -1.268 to -0.4325 | Yes | **** | <0.0001 |
| Cran 50 vs. Cran 100 | 0.1767 | -0.2410 to 0.5945 | No | ns | 0.7974 |

Cran= Cranberry; CHX= Chlorexidine

**Supplementary Figure 1**. Cluster analysis of differential expression genes. Hierarchical clustering analysis was carried out with the log10(Fragments Per Kilobase of transcript per Million mapped reads=FPKM+1) of union differential expression genes of all comparison groups under different experimental conditions. Genes in one cluster have similar expression levels. The x axis represents sample names, the y axis represents the corrected expression level value.

**Supplementary figure 2A.** TopGO DAG (Directed Acyclic Graph, DAG) of the enriched GO (Gene Ontology) term of differential expression genes and its hierarchical relation for the Control 2 (G2+) and Control 1 (G2-). Each node represents a GO term, and Top 10 GO terms are boxed. The darker the color is, the higher is the enrichment level of the term. The name and p-value of each term are present on the node.

**Supplementary figure 2B.** TopGO DAG (Directed Acyclic Graph, DAG) of the enriched GO (Gene Ontology) term of differential expression genes and its hierarchical relation for the Control 2 (G2+) and Control 1 (G2-). Each node represents a GO term, and Top 10 GO terms are boxed. The darker the color is, the higher is the enrichment level of the term. The name and p-value of each term are present on the node.

**Supplementary figure 2C.** TopGO DAG (Directed Acyclic Graph, DAG) of the enriched GO (Gene Ontology) term of differential expression genes and its hierarchical relation for the Control 2 (G2+) and Control 1 (G2-). Each node represents a GO term, and Top 10 GO terms are boxed. The darker the color is, the higher is the enrichment level of the term. The name and p-value of each term are present on the node.

**Supplementary Figure 3.** Functional annotation with EggNOG (Evolutionary Genealogy of Genes: Non-supervised Orthologous Groups) – unigene total absolute of the Collagenases U32 gene expression.

**Supplementary figure 4.** SEM images from dentine slabs after exposure to dysbiotic root caries-like biofilms and control (Control - = “G2-“, Control+sugar = “G2+”).

A


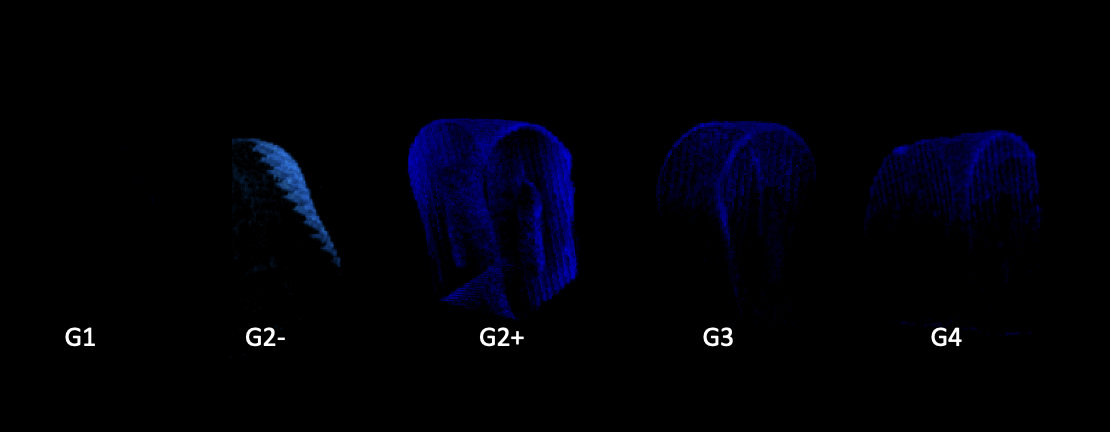


B

**Supplementary figure 5**. Examples of micro computed tomography (μ-CT) of dentine slabs confirming the presence of root caries-like lesions and mineral loss (blue areas represents the difference in the reference and target images, I.e., the demineralised area). A) 2D images for the groups exposed or not to sucrose cycles; B) 3D images for the following groups:

G1; without inoculum;

G2+; with inoculum, no pre-treatment, with sucrose cycles;

G2- ; with inoculum, no pre-treatment, no sucrose cycles;

G3; with inoculum, with LDT11 pre-treatment, with sucrose cycles;

G4; with inoculum, with cranberry pre-treatment, with sucrose cycles.
